# Ancestry-associated transcriptomic profiles of breast cancer in patients of African, Arab, and European ancestry

**DOI:** 10.1038/s41523-021-00215-x

**Published:** 2021-02-08

**Authors:** Jessica Roelands, Raghvendra Mall, Hossam Almeer, Remy Thomas, Mahmoud G. Mohamed, Shahinaz Bedri, Salha Bujassoum Al-Bader, Kulsoom Junejo, Elad Ziv, Rosalyn W. Sayaman, Peter J. K. Kuppen, Davide Bedognetti, Wouter Hendrickx, Julie Decock

**Affiliations:** 1 Functional Cancer Omics Lab, Cancer Group, Research Branch, Sidra Medicine, Doha, Qatar; 2 Department of Surgery, Leiden University Medical Center, Leiden, Netherlands; 3 Qatar Computing Research Institute (QCRI), Hamad Bin Khalifa University (HBKU), Qatar Foundation (QF), Doha, Qatar; 4 Cancer Research Center, Qatar Biomedical Research Institute (QBRI), Hamad Bin Khalifa University (HBKU), Qatar Foundation (QF), Doha, Qatar; 5 Women’s Hospital, Hamad Medical Corporation, Doha, Qatar; 6 Department of Internal Medicine and Medical Specialties (DiMI), University of Genoa, Genoa, Italy; 7 Weill Cornell Medicine-Qatar, Doha, Qatar; 8 National Center for Cancer Care and Research (NCCCR), Hamad General Hospital, Doha, Qatar; 9 General Surgery Department, Hamad General Hospital, Doha, Qatar; 10 Department of Medicine, Institute for Human Genetics, Helen Diller Family Comprehensive Cancer Center, University of California, San Francisco, CA USA; 11 Department of Population Sciences, Beckman Research Institute, City of Hope Comprehensive Cancer Center, Duarte, CA USA; 12 Department of Laboratory Medicine, Helen Diller Family Comprehensive Cancer Center, University of California, San Francisco, CA USA; 13 Cancer Immunogenetics Lab, Cancer Group, Research Branch, Sidra Medicine, Doha, Qatar; 14 College of Health and Life Sciences (CHLS), Hamad bin Khalifa University (HBKU), Qatar Foundation (QF), Doha, Qatar

**Keywords:** Prognostic markers, Oncogenesis, Breast cancer, Cancer genomics, Tumour immunology

## Abstract

Breast cancer largely dominates the global cancer burden statistics; however, there are striking disparities in mortality rates across countries. While socioeconomic factors contribute to population-based differences in mortality, they do not fully explain disparity among women of African ancestry (AA) and Arab ancestry (ArA) compared to women of European ancestry (EA). In this study, we sought to identify molecular differences that could provide insight into the biology of ancestry-associated disparities in clinical outcomes. We applied a unique approach that combines the use of curated survival data from The Cancer Genome Atlas (TCGA) Pan-Cancer clinical data resource, improved single-nucleotide polymorphism-based inferred ancestry assignment, and a novel breast cancer subtype classification to interrogate the TCGA and a local Arab breast cancer dataset. We observed an enrichment of BasalMyo tumors in AA patients (38 vs 16.5% in EA, *p* = 1.30E − 10), associated with a significant worse overall (hazard ratio (HR) = 2.39, *p* = 0.02) and disease-specific survival (HR = 2.57, *p* = 0.03). Gene set enrichment analysis of BasalMyo AA and EA samples revealed differences in the abundance of T-regulatory and T-helper type 2 cells, and enrichment of cancer-related pathways with prognostic implications (AA: PI3K-Akt-mTOR and ErbB signaling; EA: EGF, estrogen-dependent and DNA repair signaling). Strikingly, AMPK signaling was associated with opposing prognostic connotation (AA: 10-year HR = 2.79, EA: 10-year HR = 0.34). Analysis of ArA patients suggests enrichment of BasalMyo tumors with a trend for differential enrichment of T-regulatory cells and AMPK signaling. Together, our findings suggest that the disparity in the clinical outcome of AA breast cancer patients is likely related to differences in cancer-related and microenvironmental features.

## Introduction

As we enter an era of personalized medicine in oncology, large-scale studies have been instrumental in deciphering the pathogenesis and evolution of tumors. Public data repositories such as The Cancer Genome Atlas (TCGA) have enabled researchers to define the genomic landscape of different types of cancers, including breast cancer. The public availability of large-scale datasets has led to a surge in candidate drug targets and novel prognostic and/or predictive gene signatures. However, it is important to note that the majority of patients in public datasets are of European ancestry (EA), and, hence, the knowledge gained from such studies might not be applicable to patients of a different ancestry^1^. Given the global disparities in clinical behavior of breast cancer, it has become imperative to investigate ancestry-associated differences in tumor biology.

Breast cancer in women of African ancestry (AA) presents at a younger age, and is associated with more advanced disease and higher mortality rates as compared to breast cancer in age-matched patients of EA or Asian ancestry (AsA)^2–10^. Several reports have demonstrated an increased frequency of the more aggressive triple-negative breast cancer (TNBC) subtype and of the PAM50-molecular basal subtype in AA women^7–16^. Moreover, African-American women with early-stage TNBCs have been shown to exhibit a lower pathological complete response to neoadjuvant chemotherapy^17^. Interestingly, this discrepancy in clinical outcome remains after correcting for socioeconomic factors, suggesting the presence of molecular differences by ancestry^18,19^. The African-American breast cancer epidemiology and risk consortium identified few rare germline single-nucleotide polymorphisms (SNPs) that are associated with an increased risk of hormone receptor-negative breast cancer and/or TNBC in African-American women^20,21^. Analysis of genotypic traits revealed that most somatic mutations and copy number variations are subtype-specific rather than ancestrally determined^22,23^. Very few mutations showed dissimilar frequencies across African, African-American, or European-American patient subgroups when considering a specific breast cancer subtype. Likewise, numerous differentially expressed genes have been identified between breast tumors of patients of AA and EA^24–28^; however, there is little to no evidence linking these findings to differences in breast cancer survival or subtype-specific survival in relation to ancestry. Therefore, differential expression of genes involved in biological processes such as differentiation, cell cycle, DNA repair, invasion, metastasis, and angiogenesis could be related to the higher proportion of triple-negative breast tumors in the African-American population. To address this, several studies investigated molecular differences within TNBC tumors of African-American and European-American patients. TNBC tumors of African-American women were shown to display enrichment of gene sets related to a high proliferative rate, high genomic grade index, *BRCA1* deficiency, increased activation of insulin-like growth factor 1 receptor, and increased angiogenesis, closely resembling the basal like-1 TNBC subtype gene signature as described by Lehmann et al.^23,28–33^. In addition, it has been suggested that an abundance of cancer stem cells might, in part, contribute to the worse survival of African-American women with TNBC tumors^34–38^.

Given the importance of immune cell infiltration in determining the prognosis and treatment response of breast cancer, and, especially in TNBC, it is important to investigate whether differences in antitumor immunity may contribute to the divergent clinical behavior of breast cancer across populations^39–42^. To date, this phenotypic aspect of breast cancer is largely unexplored in the context of ancestry. Interestingly, systemic levels of pro-inflammatory cytokines such as interferon-γ and interleukin-6 have been found to be elevated in both healthy African-American women and those affected with breast cancer as compared to European-American women, suggesting ancestry-inferred differences in the immune response that might affect antitumor immunity and ultimately breast cancer clinical outcome^43,44^. In contrast, only subtle differences in immune gene signatures related to immune cell infiltration were found in TNBC tumors of women of AA^22,45^.

In this study, we applied a unique approach to explore ancestry-associated heterogeneity of breast cancer outcomes. First, we used improved and curated survival information from the TCGA Pan-Cancer clinical data resource (TCGA-CDR)^46^. Second, we applied SNP-based inference of ancestry^47,48^ to improve ancestry assignment, enabling us to include a substantial number of additional patients from the TCGA dataset in our analysis, thereby increasing the power of our study. Third, we performed a comprehensive transcriptomic analysis of both immunological and cancer cell-intrinsic parameters within breast cancer subtypes as defined by a novel PAM50 classification. This refined classifier utilizes a combination of Topological Data Analysis (TDA) signatures of normal mammary cell types (basal epithelial cells, luminal epithelial cells, myoepithelial cells, and Her2-related expression) to subgroup breast tumors into seven distinct molecular subtypes with prognostic value^49^. Using this combined novel approach, we interrogated the TCGA breast cancer dataset, comprising of patients of AA (*n* = 184), EA (*n* = 811), and AsA (*n* = 56), and a local Arab/Asian breast cancer dataset from Qatar (*n* = 24) for ancestry-specific molecular differences in breast cancer.

## Results

### Ancestry of patient populations

To date, studies investigating molecular differences between ancestries have been solely based on self-identified ancestry. In our study, we applied a novel approach combining self-reported ancestry and SNP-based inference of ancestry^47,48^. Ancestries were assigned using principal component (PC) analysis of SNP array genotyping calls following the method as described by Carrot-Zhang et al.^48^ (Supplementary Fig. 1). As such, we included 1051 patients from the TCGA breast cancer dataset in our analysis, of which 811 EA, 184 AA, and 56 AsA patients (Table 1). Ancestry of patients in the local Retrospective Arab cohort from Qatar (RA-QA) was solely based on self-reported ancestry, subgrouping 16 patients as Arab ancestry (ArA), five as AsA, two as EA, and one as Persian (Table 2).

**Table 1:** Cohort demographics of the TCGA breast cancer cohort.

TCGA-BRCA cohort (*n* = 1082)		
Median FU (years)	2.37	
Events		
OS	151	
DSS	83	
Age (years)		
Median	58	
Range	26–90	
	*n*	%
Ancestry^a^		
European	811	75
African	184	17
Asian	56	5.2
Undefined	31	2.9
AJCC stage		
I	179	16.8
II	613	56.6
III	247	22.7
IV	19	1.8
NA	24	2.2
PAM50 subtype		
Basal	233	22
Her2-enriched	160	14
Luminal A	337	31
Luminal B	241	22
Normal-like	111	10
TDA subtype		
BasalHer2	82	8
BasalMyo	219	20
BasalLumHer2	90	8
Lum	283	26
LumBasal	209	19
MyoLumA	102	9
MyoLumB	35	3
MyoLumHer2	62	6

^a^SNP-based ancestry

**Table 2:** Cohort demographics of RA-QA breast cancer cohort.

RA-QA cohort (*n* = 24)		
Median FU (years)	8.02	
Events		
OS	7	
Age (years)		
Median	48.5	
Range	28–63	
	*n*	%
Ancestry^a^		
Arab	16	66.7
Asian	5	20.8
Caucasian	2	8.4
Persian	1	4.2
AJCC stage		
I	4	16.7
II	10	41.7
III	4	16.7
IV	0	0
NA	6	25
PAM50 subtype		
Basal	9	37.5
Her2-enriched	3	12.5
Luminal A	7	29.2
Luminal B	2	8.3
Normal-like	3	12.5
TDA subtype		
BasalHer2	2	8.3
BasalMyo	7	29.2
BasalLumHer2	2	8.3
Lum	6	25
LumBasal	2	8.3
MyoLumA	1	4.2
MyoLumB	1	4.2
MyoLumHer2	3	12.5

^a^Self-reported ancestry

### Distribution of molecular breast cancer subtypes

**Fig. 1:**
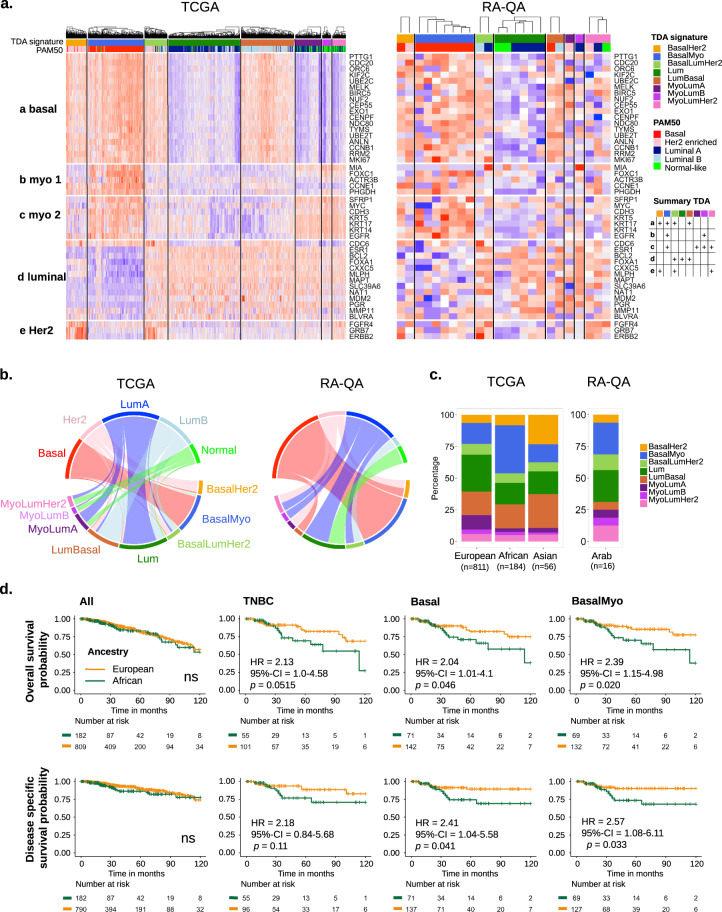
Distribution of breast cancer molecular subtypes defined by topological data analysis (TDA) signatures across ancestries. **a** Heatmap of expression of PAM50 genes organized by TDA signature classes in TCGA breast cancer and RA-QA cohort. Samples are annotated by TDA signature class (upper annotation bar) and classical PAM50 intrinsic molecular subtype (lower annotation bar). The combination patterns of upregulated expression of five distinct gene groups defining each TDA class are summarized in a table on the right (Summary TDA). **b** Reclassification of breast cancer samples from classical PAM50 intrinsic molecular subtypes (upper part of circos) to TDA signature classes (lower part of circos) in TCGA and RA-QA breast cancer cohorts. **c** Stacked bar chart of distribution of TDA classes by ancestry. **d** Kaplan–Meier plots showing overall survival (upper panels) and disease-specific survival (lower panels) by ancestry. Difference between the survival of patients with European and African ancestry is shown for the complete TCGA breast cancer cohort (left), patients with TNBC according to hormone receptor status (middle left), patients with PAM50-defined basal breast cancer (middle right), and patients with tumors classified as BasalMyo by TDA classification (right). Censor points are indicated by vertical lines.

Numerous studies have demonstrated a higher prevalence of TNBC and of tumors of the molecular basal subtype among AA women and have linked the increased frequency of these aggressive breast tumors to ancestry-associated disparity in breast cancer clinical outcome. Using our novel combined approach, we interrogated the TCGA and RA-QA datasets to subgroup patients according to TDA-defined molecular subtype and ancestry^49^. Heatmaps of TCGA and RA-QA samples based on TDA gene signatures (basal, myo1, myo2, luminal, and Her2) show a clear segregation of samples in seven molecular subtypes, each defined by a unique combination of expression of five distinct gene groups, demonstrating the accuracy and robustness of the novel classifier (Fig. 1a). As can be seen in the circos plots in Fig. 1b, and in accordance with the METABRIC analysis by Mathews et al.^49^, we found that luminal A tumors are mainly reclassified into Lum and MyoLum subgroups, while luminal B tumors are mainly subgrouped into LumBasal and Lum tumors. In addition, tumors of the normal-like PAM50 subtype are mainly reclassified into the Myo classes. Her2-enriched tumors are predominantly subdivided into BasalHer2, BasalLumHer2, and LumBasal tumors. Further, the vast majority of basal tumors are reclassified as BasalMyo (88%). Figure 1c clearly demonstrates differences in molecular subtype frequency across ancestries, with a strong enrichment in AA patients of BasalMyo (38.0 vs 16.5% in EA, *χ*^2^ = 41.3, *p* = 1.30E − 10) and a reduced proportion of MyoLumA (2.7 vs 11% in EA, *χ*^2^ = 11.7, *p* = 0.0006) and Lum (17 vs 29% in EA, *χ*^2^ = 10.9, *p* = 0.001) tumors, and in AsA patients an enrichment of BasalHer2 tumors (21.7 vs 6.4% in EA, *χ*^2^ = 19.0, *p* = 1.33E − 05). While several studies reported an increase in basal tumors with worse outcome in AA patients^7,9,11,12,29,50^, we were able to fine-tune this observation to a strong increase of BasalMyo tumors, accounting for the majority of basal tumors. Furthermore, we observed an increase in the proportion of BasalMyo tumors in ArA patients (25.0 vs 16.5% in EA, *χ*^2^ = 1.0E − 4, n.s.), although this did not reach statistical significance as a likely result of the small cohort size.

Next, we explored ancestry-related differences in clinical outcome using curated survival data from the TCGA-CDR^46^. The clinical outcome of breast cancer patients, irrespective of molecular subtype, was not different between EA and AA patients (Fig. 1d). Among all seven TDA subtypes, BasalMyo tumors were the only tumors that were associated with significantly different 10-year overall survival (OS, *p* = 0.020) and disease-specific survival (DSS, *p* = 0.033) rates for AA vs EA patients (Fig. 1d and Supplementary Fig. 2). The 5-year OS rates for BasalMyo tumors were 85.5% for EA and 70.1% for AA patients (*p* = 0.07), and the 5-year DSS rates were 90.1% for EA and 73.6% for AA patients (*p* = 0.05). Interestingly, compared to TNBC and basal tumors, we observed a larger disparity in 10-year OS (hazard ratio (HR) = 2.39, *p* = 0.020) and 10-year DSS (HR = 2.57, *p* = 0.033) by ancestry in BasalMyo tumors (Fig. 1d). To exclude that this survival difference results from a higher frequency of more advanced stage BasalMyo tumors in AA patients, we compared the AJCC pathological stage between EA and AA patients and found no significant difference in stage distribution by ancestry (*χ*^2^ = 2.83, *p* = 0.092) (Supplementary Fig. 3). In addition, we performed survival analysis stratified by early (stages I and II) and advanced (stages III and IV) stage and found rather large HRs, although not significant, indicating worse OS of AA patients within strata (Supplementary Fig. 3). Adjustment for tumor stage and/or age in multivariate analysis showed similar results with AA being associated with worse survival (Supplementary Fig. 3), albeit with borderline significance, implying that additional factors beyond pathological stage contribute to the divergent clinical outcome of AA patients with BasalMyo tumors compared to EA patients.

### Ancestry-associated differences in immunological parameters

In an effort to elucidate potential ancestry-inferred differences in tumor biology, we compared the immune microenvironment of tumors from patients with different ancestry. More specifically, we assessed tumor immune disposition using the prognostic Immunologic Constant of Rejection (ICR) immune gene signature^51,52^ and deconvoluted immune cell abundance using leukocyte subgroup enrichment scores (LES)^53^. The ICR 20-gene signature consists of genes encoding CXCR3/CCR5 chemokine ligands (*CXCL9*, *CXCL10*, and *CCL5*), genes encoding molecules involved in T-helper type 1 (Th1) signaling (*IFNG*, *TXB21*, *CD8B*, *CD8A*, *IL12B*, *STAT1*, and *IRF1*), and effector immune functions (GNLY, PRF1, GZMA, GZMB, and GZMH), as well as counter-regulatory molecules (IDO1, PDCD1/PD-1, CD274/PD-L1, CTLA4, and FOXP3). Using the ICR gene signature, we previously classified breast cancer samples into four classes with the highest activation of the antitumor immune response in the ICR4 class^51^. In a follow-up study of >8000 nonmetastatic breast cancer cases, we demonstrated that the ICR signature was the strongest independent prognostic predictor for metastatic relapse, in particular for patients with Her2+-enriched and triple-negative breast tumors^54^. Since we did not consider ancestry in our previous findings, the present study aimed to investigate whether the prognostic value of ICR holds true across ancestries or whether there could be immune-related dysregulations that, in part, explain the disparity in the clinical outcome of AA breast cancer patients. First, we used the ESTIMATEscore, ImmuneScore, and StromalScore to compare tumor cellularity, proportion of the stromal component, and level of infiltration of immune cells of all TDA subtypes in EA vs AA patients^55^. We did not observe significant differences within subtypes by ancestry, indicating that any potential changes in immune-related gene expression in AA vs EA patients are not caused by differences in stromal and immune cell composition (Supplementary Fig. 4).

**Fig. 2:**
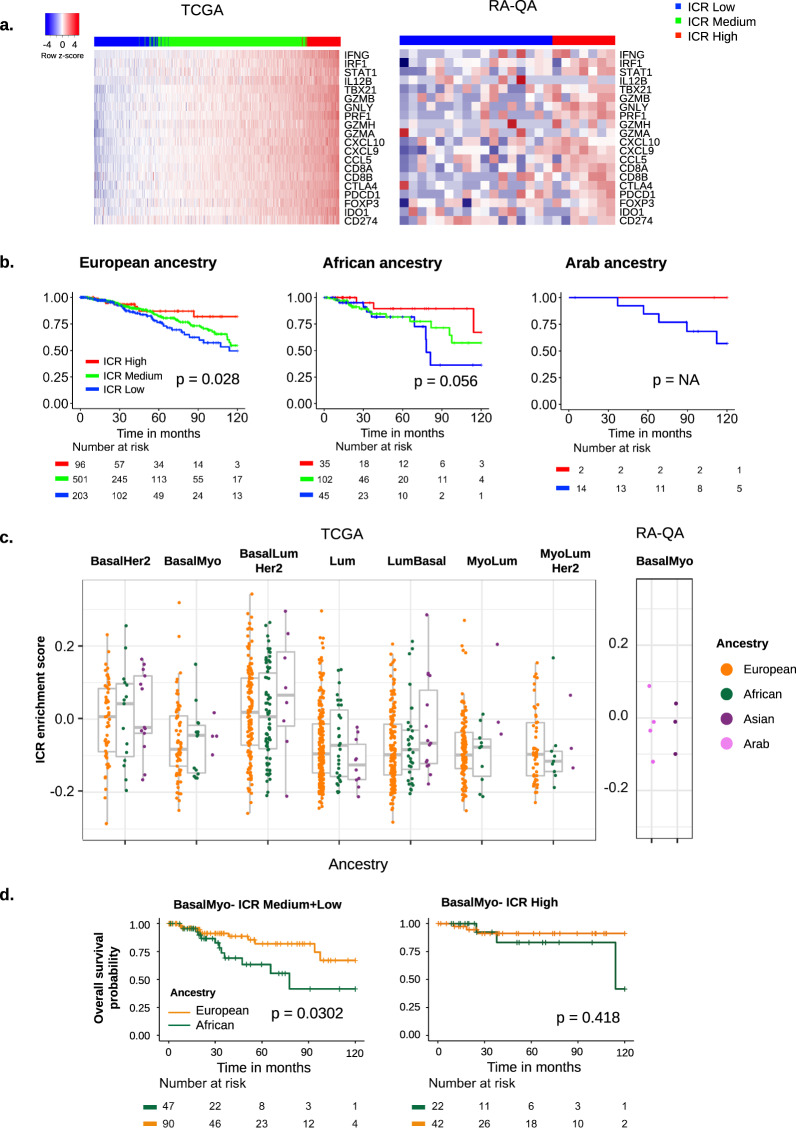
Tumor immune phenotypes and clinical outcome by ancestry. **a** Heatmap of ICR gene expression in TCGA and RA-QA breast cancer cohorts. Classification of samples by ICR consensus clustering segregates TCGA samples in ICR low, ICR medium, and ICR high groups. Samples of RA-QA cohort were classified as ICR low or ICR high. **b** Kaplan–Meier plots showing overall survival across ICR groups in breast cancer TCGA patients of EA (left), TCGA patients of AA (middle), and RA-QA patients of ArA (right). **c** ICR enrichment scores across ancestries within TDA signature classes. Box plots indicate medians and interquartile range, and whiskers represent 10th and 90th percentile. All data points are plotted individually. **d** Overall survival of EA and AA patients in TCGA BasalMyo samples classified as ICR medium + low (left), and ICR high (right). Censor points are indicated by vertical lines.

The ICR gene signature clearly clusters breast tumors of the TCGA dataset into three immune phenotypes with varying degrees of immune activation (ICR low, ICR medium, and ICR high), while tumors of the RA-QA cohort were subdivided into two immune phenotypes (ICR low and ICR high) (Fig. 2a). In accordance with our previous work, tumors with an ICR Low immune phenotype were associated with a worse survival in EA patients (*p* = 0.028) (Fig. 2b). Likewise, we observed a large, although not significant, difference in survival between ICR low and ICR high patients within the AA and ArA groups. In line with these findings, the prognostic value of gene signatures that reflect the abundance of individual immune cell populations was overall similar across ancestries with leukocyte subpopulations classically associated with better prognosis such as CD8+ T cells and cytotoxic cells having the same trends in EA and AA patients (Supplementary Fig. 5). Next, we investigated whether the immune disposition, inferred from the ICR enrichment score, varies within TDA subtypes by ancestry (Fig. 2c). Comparison of the continuous ICR enrichment score demonstrated modest variation between TDA subtypes with overall higher scores in non-luminal tumors (BasalHer2 and BasalMyo), which was not affected by ancestry. For instance, no significant difference in ICR enrichment score was found in BasalMyo tumors by ancestry, suggesting a similar overall immune disposition across ancestries. In accordance, we did not find any significant differences in the expression of individual ICR genes based on ancestry (data not shown). Further analysis of BasalMyo tumors, however, revealed differences within ICR clusters whereby ICR low and ICR medium patients were grouped into one subgroup due to the limited sample size of each cluster within BasalMyo tumors. Although BasalMyo tumors of AA patients were overall associated with worse OS, this was more pronounced in ICR medium + low tumors (10-year OS, *p* = 0.03; 5-year OS, *p* = 0.07) (Fig. 2d). In multivariate analysis, AA remained significantly associated with worse survival when adjusted for tumor stage, and reached borderline significance when adjusted for tumor stage and age (Supplementary Fig. 3).

**Fig. 3:**
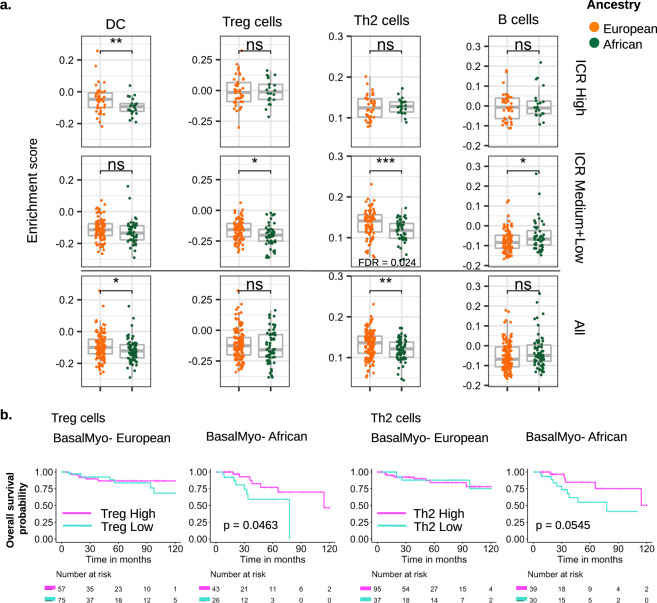
Enrichment of immune cell subpopulations in AA and EA patients with BasalMyo breast tumors. **a**. Enrichment scores of signatures reflecting the abundance of dendritic cells (DCs), T-regulatory cells (Tregs), T-helper 2 (Th2), and B cells in BasalMyo tumor samples of EA and AA patients. Box plots are facetted by ICR groups, ICR high (upper panels), ICR medium + low (middle panels), and across all samples (lower panels). Box plots indicate medians and interquartile range, and whiskers represent 10th and 90th percentile. All data points are plotted individually. *T* test (two-sided): *p < 0.05, **p < 0.01, ***p < 0.001, and n.s. not significant. Adjusted *p* value (FDR) by Benjamini and Hochberg method. **b** Kaplan–Meier plots of overall survival in EA and AA patients with BasalMyo breast cancer dichotomized by enrichment scores of TReg (left panels) and Th2 cell signatures (right panels). Cutoff for dichotomization in “High” and “Low” categories is based on optimal enrichment cutoff determined by XGBoost model used for survival analysis. Censor points are indicated by vertical lines.

This finding raised the question whether the worse outcome of AA patients with BasalMyo tumors is linked to molecular differences in ICR medium + low tumors also known as cold tumors. For this purpose, we determined the LES of 24 distinct immune cell types (Fig. 3a). Focused analysis of BasalMyo cold (ICR medium + low) tumors revealed a significant decrease in T-regulatory cell (Tregs) and Th2 enrichment scores (*p* = 0.036; *p* = 3.36E − 4, respectively), and a small increase in B cell enrichment score (*p* = 0.039) in AA vs EA patients, whereas dendritic cell (DC) enrichment scores were reduced in ICR hot (ICR high) tumors (*p* = 0.009).

In order to identify which LES may harbor prognostic value, we focused on BasalMyo tumors irrespective of ICR class due to sample size limitations and adopted a machine-learning strategy, which has empirically been shown to work efficiently on small size datasets^56–58^, despite a slight tendency for overfitting (EA, *n* = 134; AA, *n* = 70). First, we performed a sensitivity model analysis that enabled us to identify the XGboost models that have an optimal set of hyper-parameters (Harrell’s C index EA = 0. 58, AA = 0.63) with relatively small variance (data not shown). Next, we used XGBoost modeling for nonlinear multivariate Cox regression survival analysis followed by the SHapley Additive exPlanation (SHAP) method for the AA and EA subgroups separately (Supplementary Fig. 6). This approach provided information on which features or gene signatures are the most important and their range of effects over the dataset, including the breadth (SHAP value) and the direction of the effect (positive or negative). Both the Treg and Th2 signature were classified as features with more importance for predicting outcome in AA patients as compared to outcome in EA patients, with reduced enrichment scores being associated with increased risk of death. In accordance, we found that AA, but not EA, patients could be stratified into different risk groups based on the expression of the Treg and Th2 cell signatures with borderline statistically different clinical outcomes (Fig. 3b). More specifically, stratification by Treg LES subgrouped AA patients with BasalMyo tumors in a low-risk group with higher expression and 5-year OS rate of 77%, and a high-risk group with low expression and 5-year OS rate of 59% (10-year HR = 2.99, 95% confidence interval (CI) = 1.02–8.77). Th2 LES-based stratification grouped AA patients with BasalMyo tumors into a low-risk/high expression group with 5-year survival rate of 84% and a high-risk/low expression group with 5-year survival rate of 55% (10-year HR = 3.13, 95% CI = 0.98–10.00). No differences in survival were noted for DC and B cell LES (data not shown), which supports their lower rank of importance in the SHAP plot of AA patients (Supplementary Fig. 6).

### Ancestry-associated differences in cancer cell-intrinsic features

Next, we investigated whether specific cancer cell-intrinsic features might contribute to the worse survival of AA patients with BasalMyo tumors. First, we examined potential changes in common cancer-associated genomic aberrations, including mutational load, neoantigen load, and tumor aneuploidy. Remarkably, non-silent mutation rate was significantly lower in AA patients compared to EA (*p* = 0.025), while the number of predicted single-nucleotide variant neoantigens was similar between both patient populations (Supplementary Fig. 7). Therefore, we speculated that AA BasalMyo tumors undergo less immunoediting and immune-mediated elimination of neoantigens compared to EA BasalMyo tumors. To address this hypothesis, we used an “immunoediting score,” defined as the observed ratio (number of point mutations predicted to generate neo-epitopes divided by the total count of non-silent point mutations) compared to the expected ratio (expected numbers based on silent mutation rate)^59^. Indeed, the ratio of the observed/expected neoantigens was increased in AA patients (*p* = 0.033), suggesting reduced immunoediting in AA samples (Supplementary Fig. 7). However, we did not observe any survival difference between tumors with a high observed/expected neoantigen ratio compared to tumors with a low ratio (HR = 1.1, 95% CI = 0.43–2.79, *p* = 0.842), suggesting that this tumor attribute does not explain the observed survival differences between AA and EA BasalMyo tumors. Similarly, while we observed a significantly increased tumor aneuploidy score in samples of AA patients (*p* = 0.008, Supplementary Fig. 7), this tumor characteristic was not associated with a difference in survival (HR = 0.691, 95% CI = 0.32–1.48, *p* = 0.34).

**Fig. 4:**
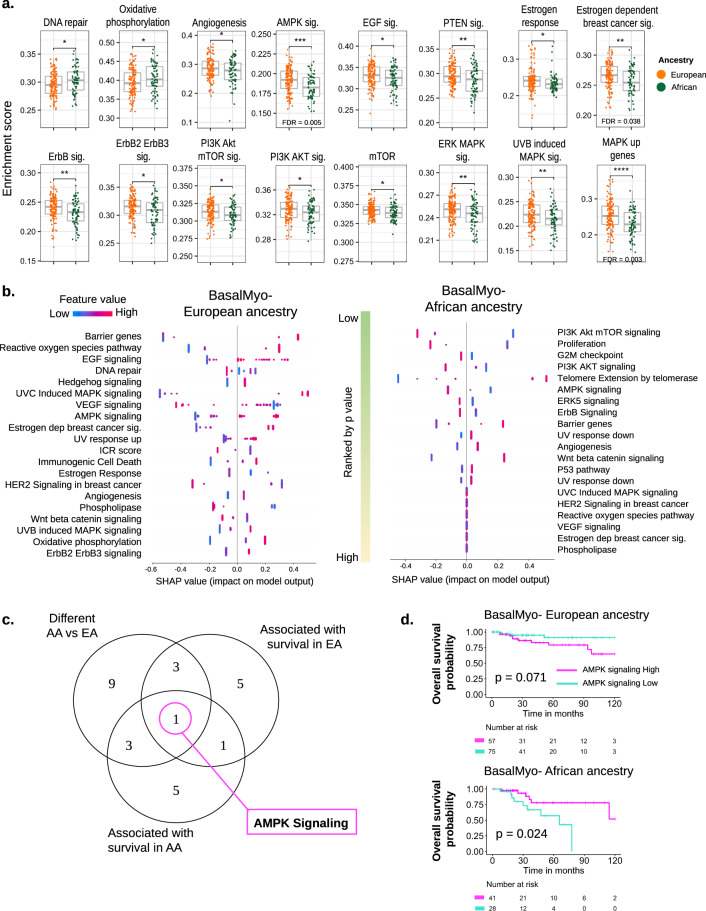
Differentially enriched oncogenic pathways with prognostic connotation in EA and AA patients with BasalMyo breast tumors. **a** Enrichment scores of signatures of tumor-associated pathways that are differentially regulated between EA and AA patients with BasalMyo tumors. Box plots indicate medians and interquartile range, and whiskers represent 10th and 90th percentile. All data points are plotted individually. *T* test (two-sided): *p < 0.05, **p < 0.01, ***p < 0.001, and ****p < 0.0001. Adjusted *p* value (FDR) by Benjamini and Hochberg method. **b** SHAP plots of tumor-associated pathways that are associated with overall survival in EA (left) and AA (right) patients with BasalMyo breast tumors. Pathways are ranked by *p* value to reflect the importance of each feature in the survival model. Each dot represents a single sample and is colored by relative enrichment score. Corresponding impact on model output (SHAP value) ranges from −1 (indicating the absence of an event) to +1 (indicating the occurrence of an event, in this case, death). **c** Intersection of differentially enriched tumor-associated pathways with ten most important pathways in AA and EA patients with BasalMyo breast tumors. AMPK signaling is differentially regulated in AA vs EA and is of importance in survival models of both AA and EA patients. **d** Kaplan–Meier curves visualizing the prognostic value of AMPK signaling in EA (upper) and AA (lower) BasalMyo patients. Dichotomization of samples by AMPK signaling is based on optimal enrichment score cutoff as determined by XGBoost model. Censor points are indicated by vertical lines.

To further explore tumor intrinsic features that could contribute to the divergent survival outcomes, we explored the differential enrichment of 54 cancer-associated pathways (Fig. 4a). A total of 16 pathways were found to be differentially enriched between BasalMyo tumors of AA vs EA patients. Of note, only 2 out of 16 pathways, DNA repair and oxidative phosphorylation, were associated with an increased enrichment in AA patients. A number of enriched pathways were identified multiple times as they were included in more than one database, including estrogen response and estrogen-dependent breast cancer signaling, ErbB signaling and ErbB2/ErbB3 signaling, PI3K-Akt mTOR signaling and PI3K-AKT signaling or mTOR signaling, and ERK MAPK signaling, ultraviolet B (UVB)-induced MAPK signaling, and MAPK up genes. Furthermore, the pathways defined as angiogenesis, AMPK signaling, EGF signaling, and PTEN signaling were significantly less enriched in BasalMyo tumors of AA vs EA patients. Using the same approach that we applied to explore the prognostic value of immune gene signatures, we used XGBoost modeling and the SHAP method to identify which cancer-associated pathways are the most powerful indicators of poor survival in AA vs EA patients with BasalMyo tumors (Fig. 4b, c). Based on the summary SHAP plots, we observed that among the top 10 pathways affecting survival in EA patients, the majority displayed an inverse correlation of enrichment with survival, including barrier genes, reactive oxygen species pathway, EGF signaling, hedgehog signaling, UVC-induced MAPK signaling, AMPK signaling, estrogen-dependent breast cancer signaling, and UV response up genes (Fig. 4b). In contrast, increased enrichment of DNA repair and VEGF signaling pathways were associated with better survival in EA patients. In AA patients, the majority of the top 10 pathways determining survival exhibited better survival with increased enrichment including PI3K-Akt mTOR signaling, proliferation, G2M checkpoint, PI3K-AKT signaling, AMPK signaling, ERK5 signaling, and ErbB signaling (Fig. 4b). On the other hand, we found that pathway enrichment for telomere extension by telomerase, barrier genes, and UV response down corresponded to worse survival.

In analogy with our analysis of the prognostic value of enriched immune gene signatures, we performed a combined analysis of differentially enriched pathways and the top ten pathways with importance for the prediction of survival (Fig. 4c). Using this approach, we identified three differentially enriched pathways with prognostic value in EA patients with higher enrichment of EGF signaling (*p* = 0.02, optimal enrichment cutoff = 0.334) and estrogen-dependent breast cancer signaling (*p* = 0.076, optimal enrichment cutoff = 0.268) being associated with worse prognosis, while a better survival was observed for enrichment of DNA repair (*p* = 0.03, optimal enrichment cutoff = 0.304). Focusing on AA patients, we found three differentially enriched pathways with prognostic connotation whereby enrichment of PI3K-Akt-mTOR signaling (*p* = 9.00E − 04, optimal enrichment cutoff = 0.307), PI3K-Akt signaling (*p* = 0.006, optimal enrichment cutoff = 0.328), and ErbB signaling (*p* = 0.053, optimal enrichment cutoff = 0.232) was associated with better outcome (Fig. 4b, c). Interestingly, we found AMPK signaling to be the sole pathway to be differentially enriched between BasalMyo tumors of AA and EA patients with prognostic value in patients of both ancestries. Further analyses revealed an inverse correlation of AMPK enrichment with OS in AA vs EA patients. While in EA patients, pathway enrichment was associated with worse survival, it bestowed a survival advantage for AA patients (Fig. 4d). The 5-year OS rate of EA patients with BasalMyo tumors enriched for AMPK signaling was reduced by 12% from 91 to 79% (10-year HR = 0.343, 95% CI = 0.11–1.10), while the opposite was observed in AA patients where the 5-year OS rate was increased by 21% from 57 to 78% (10-year HR = 3.598, 95% CI = 1.18–10.94).

### Molecular alterations in Arab breast cancer patients

**Fig. 5:**
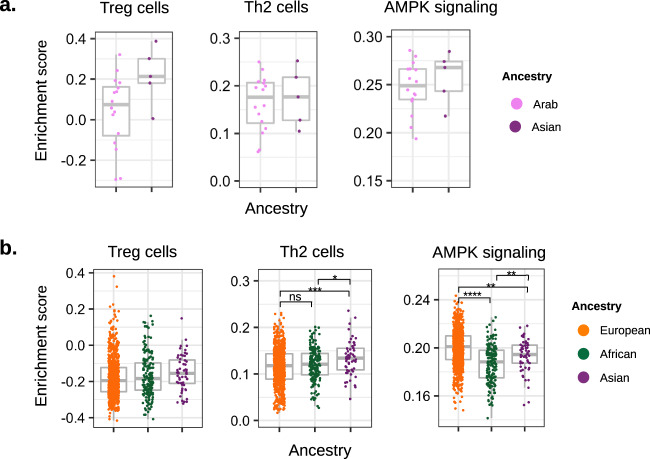
Enrichment of selected immune cell subpopulations and oncogenic pathways in Arab breast cancer patients. Enrichment scores for signatures for T-regulatory cells (Tregs, left), T-helper 2 cells (Th2, middle), and AMPK signaling (right) in panel (**a**). RA-QA cohort comparing ArA to AsA breast cancer patients, independent of molecular subtype. **b** TCGA breast cancer cohort comparing EA, AA and AsA breast cancer patients, independent of intrinsic molecular subtype. Box plots indicate medians and interquartile range, and whiskers represent 10th and 90th percentile. All data points are plotted individually. *T* test (two-sided): *p < 0.05, **p < 0.01, ***p < 0.001, ****p < 0.0001, and ns, not significant.

Given the similarity in TDA subtype distribution of ArA and AA patients (Fig. 1c), we investigated whether the increased frequency of BasalMyo tumors in ArA patients was associated with differential enrichment of LES and cancer-associated pathways. Specifically, we focused our analyses on Treg, Th2, and AMPK signaling signatures that showed differentially enrichment with prognostic value in AA patients. Due to limited cohort size, we assessed enrichment patterns in all Arab patients without subgrouping by TDA subtype. Compared to AsA patients, ArA patients showed a trend towards lower enrichment scores of the Treg and AMPK signature (Fig. 5a). In order to compare patterns of enrichment between ancestries of both cohorts, we performed a similar analysis across TCGA ancestries (EA, AA, and AsA) without TDA subgrouping (Fig. 5b). Out of the three signatures, only the differential enrichment of AMPK signaling holds true when comparing the overall AA vs EA patient population. Since BasalMyo tumors constitute a large proportion of breast tumors in the AA patients (38%) and are associated with a strong reduction in AMPK signaling (*p* = 1.78E − 04), we cautiously speculate that the overall reduced enrichment of AMPK signaling in AA patients might be related to our findings in BasalMyo tumors. Similarly, it could be plausible that our findings in Arab patients might be related to differential enrichment signatures in BasalMyo tumors, supporting the need for larger Arab patient cohorts to enable statistically powered subanalysis of TDA subgroups.

## Discussion

An increasing effort is expended to decipher the molecular differences that are associated with global disparities in breast cancer outcomes. Several studies have investigated the presentation of breast tumors in patients of African origin in comparison to women of European origin. A consensus across studies is that women of AA display a higher prevalence of the unfavorable TNBC subtype and of the molecular PAM50-defined basal subtype^7–15,60^. We interrogated the TCGA breast cancer cohort using curated survival data, improved ancestry assignment, and a refined classifier that reclassifies breast tumors into seven subgroups using the PAM50 signature in combination with TDA. Comparison of the classical PAM50 and the TDA classifier revealed that the large majority of basal tumors belong to the BasalMyo TDA subgroup, and that the reported enrichment of basal tumors in patients of AA is largely dominated by the BasalMyo subtype. Moreover, we were able to demonstrate that BasalMyo tumors are the only TDA subgroup that is associated with an ancestry-associated disparity in clinical outcome, underlining the clinical relevance of BasalMyo tumors in African patients.

In order to elucidate the underlying biological processes contributing to the worse survival of AA patients with BasalMyo tumors as compared to EA patients, we assessed transcriptomic differences in immunological parameters and cancer cell-intrinsic features. To date, only a few population-based studies have considered ancestry-related changes in the immune response of breast cancer patients^22,43–45^. Overall, very few immunological differences in tumor tissues have been reported between patients of AA and EA^22,45^. Pitt et al.^22^ reported subtle differences in tumor immune signatures when adjusting for PAM50-defined subtype. They found an enrichment of the type I IFN signature in luminal A and luminal B tumors of patients of AA, including African-American and Nigerian women, as compared to patients of EA. A study by O’Meara et al.^45^ reported no significant differences in the expression of 14 immune metagenes in TNBC tumors of AA and EA patients, whereas the proportion of resting CD4+ memory cells, as determined by CIBERSORT, was significantly higher in TNBC tumors of EA patients. Based on the notion that the CIBERSORT algorithm determines the relative abundance of immune cell subpopulations within a tumor rather than between tumors, we did not include CIBERSORT in our analyses. We explored ancestry-related differences in immune disposition using the ICR classifier of tumor immune phenotypes and LES. As such, we found that the prognostic value of the ICR immune gene signature holds true across ancestries and that the lower enrichment of Tregs and Th2 immune cells in patients of AA negatively correlated with outcome. Although this seems a counterintuitive finding, it is important to note that the presence of immunosuppressive cells could be a result of prior immune activation. In line with this, we previously found that FoxP3 expression heavily correlates with T cell infiltration as a counter-regulatory signal and hence is an important marker of the ICR signature^52^. In addition, a number of studies have reported that increased expression of immunosuppressive gene signatures supports chemotherapy sensitivity and hence better clinical outcome in (triple-negative) breast cancer^61–64^.

Subsequently, we explored whether we could identify ancestry-specific enriched oncogenic pathways with prognostic relevance in BasalMyo tumors. In support of this concept, a recent transcriptome-wide association study of the Caroline Breast Cancer Study transcriptomic dataset, comprising of self-identified African-American and European-American women, demonstrated that ancestry-stratified predictive risk models did not perform across ancestries and/or subtype^65^. Through integrative analysis of differential enrichment and prognostic connotation, we identified seven differentially enriched signaling pathways with prognostic connotation in patients of EA and/or AA. Enrichment of EGF and estrogen-dependent signaling was associated with worse clinical outcomes in patients of EA, while enrichment of DNA repair genes correlated with a better outcome. Conversely, enrichment of PI3K-Akt/PI3K-AKT-mTOR and ErbB signaling was associated with better prognosis in patients of AA. Although this survival-favorable correlation appears contradictory in relation to mTOR and ErbB-mediated oncogenic signaling, recent studies have demonstrated enrichment of PI3K-AKT signaling in immunogenic TNBC tumors, suggesting that hyperactivation of this signaling pathway might promote immunogenic activity and result in better prognosis^61,66,67^. This raises the question whether BasalMyo tumors enriched in PI3K and ErbB signaling could similarly infer an immune favorable tumor phenotype in a subset of AA patients. Furthermore, analysis of the individual molecules constituting the ErbB signaling pathway revealed a reduced enrichment of ErbB2, ErbB3, and ErbB4 and downstream signaling, irrespective of ancestry, in hormone receptor-negative tumors and in particular BasalMyo tumors compared to hormone receptor-positive tumors (data not shown). On the other hand, hormone receptor-negative tumors and BasalMyo tumors feature a higher enrichment of ErbB1/EGFR and its downstream molecules, which may be driving the overall increased enrichment of ErbB signaling in those tumors (data not shown). These findings highlight the importance of obtaining a more granular view of the changes in the ErbB pathway in BasalMyo tumors such as the relative effect of individual EGFR ligands on ErbB signaling enrichment. Notably, AMPK signaling was associated with opposing prognostic significance in EA and AA patients, with a positive connotation in the latter group. AMP-activated protein kinase or AMPK is a key regulator of cancer metabolism and oncogenic signaling, is frequently upregulated in TNBC vs non-TNBC tumors, and is generally associated with poor clinicopathological factors and shorter survival^68,69^. Several lines of evidence, however, point towards a more complex role for AMPK in cancer whereby AMPK activation has been associated with both pro-tumorigenic and anti-tumorigenic effects depending on specific metabolic cues^70^. For example, activation of AMPK signaling has been shown to inhibit the PI3K-AKT-mTOR pathway, the expression of EGFR and cyclins, and the phosphorylation of Src, STAT3, and MAPK, culminating in reduced tumorigenic potential and better clinical outcome^71–73^. It remains to be determined if metabolic-mediated dysregulation of AMPK signaling could be regulated by ancestry-specific traits. Indeed, few studies have reported ancestral disparity in cancer metabolomics^74–76^. Our finding illustrates that metabolic pathways might be governed by different regulators depending on ancestry, and hence reiterates the need to account for ancestry in biomarker and cancer target research.

To conclude, the rapidly evolving technological landscape and refinement of cancer treatment towards precision cancer medicine has led to the recognition that breast cancer is not a single disease, but should be studied and clinically managed as multiple distinct disease entities. It is now well appreciated that the complexity and heterogeneity of breast cancer arise from differences in cancer cell-intrinsic mechanisms as well as from dysregulation of the interplay with the stromal and immune microenvironment. Our findings support the notion of an additional level of complexity introduced by ancestry-associated traits and urge for more studies on underrepresented populations such as patients of ArA. Therefore, we advocate accounting for ancestry-specific molecular features in breast cancer research and in clinical decision making in order to guide precision cancer medicine.

## Methods

### Patient cohorts

Two different breast cancer cohorts were included in this study: the publicly available TCGA breast cancer dataset and a local cohort from Qatar.

RNA-sequencing data from the TCGA breast cancer cohort (*n* = 1082 patients) was downloaded using R (v3.5.1) and TCGA Assembler (v2.0.3, ref. ^77^). Sample data were extracted ensuring a single primary tumor sample per patient using the TCGA Assembler “ExtractTissueSpecificSamples” function. Clinical data for all patients were obtained from the TCGA-CDR^46^. Patient ancestry was obtained using SNP-based inferred ancestry data, focusing on the European, Asian, and African clusters^47,48^. To visualize major ancestry clusters within the TCGA-BRCA cohort, PC analysis results of Carrot-Zhang et al.^48^ were used to plot PC1 vs. PC2 using ggplot. Using these data, we were able to include 108 patients who previously had no reported ancestry. As SNP-based ancestry had a very high concordance with reported ancestry (99.1%), we decided to also include 63 patients for whom only self-reported ancestry was available. We excluded 31 patients from our ancestry-based analyses. First, 16 patients with American inferred ancestry as the number of samples in this cluster is limited as well as one patient who self-identified as not Hispanic or Latino. Second, six patients without self-reported or inferred ancestry and third exceptional cases of discordance between self-reported and SNP-based ancestry (*n* = 8; 0.9%) were excluded. The final TCGA breast cancer cohort used for analysis comprises 1051 patients (811 of EA, 184 of AA, and 56 of AsA). The tumor non-silent mutation rate, predicted neoantigen load, and aneuploidy score were obtained from Thorsson et al.^78^, and predicted vs expected neoantigen values were extracted from Rooney et al.^59^.

The RA-QA patient cohort constitutes a breast cancer cohort from Qatar (*n* = 24 of which 16 of ArA) with patients who were newly diagnosed with breast cancer between 2004 and 2010 at the National Centre for Cancer Care and Research (NCCCR) in Doha. Clinical information and self-reported ancestry were extracted from the medical records. The study was approved by the local ethical committees of the Hamad Medical Corporation (study approval number #14027/14), the Qatar Biomedical Research Institute (study approval number #2016-002), and Sidra Medicine (study approval number #1711015664), and was performed in accordance with the ethical standards of the institutional and/or national research committee and with the 1964 Declaration of Helsinki and its later amendments or comparable ethical standards.

The study protocol was granted a waiver of informed consent under the condition of anonymization and no additional intervention for the participants.

### Total RNA-sequencing

RNA was isolated from four 20 μm sections of formalin-fixed paraffin-embedded (FFPE) tumor samples of the RA-QA cohort using the AllPrep DNA/RNA FFPE kit (Qiagen, Germany), followed by a quality control for purity and integrity by the Agilent Bioanalyzer system. Total RNA was depleted from ribosomal RNA and random primed for complementary DNA synthesis using the TruSeq-stranded total RNA kit (Illumina, USA). RNA-sequencing was performed on the Illumina HiSeq2500 platform (Illumina) with paired-end 25× coverage (PE100–125). The FASTQ files were trimmed to remove adaptor sequences using flexbar (v3.0.3, ref. ^79^) and aligned to GRCh37/hg19 reference genome using hisat2 (v2.0.5, ref. ^80^), resulting in an average 10–15 M aligned reads. Reads were counted to genomic features using subreads (v1.5.5, ref. ^81^). For both the TCGA and RA-QA cohort, RNA-seq data were corrected for GC content and normalized within and between lanes using the R package EDASeq (v2.12.0, ref. ^82^), and quantile normalized using the preprocessCore (v1.36.0, ref. ^83^).

### Intrinsic molecular subtype classification

The intrinsic molecular subtype of each tumor sample was defined by the differential expression of a set of 50 genes (PAM50) using two distinct algorithms. First, the R package bioclassifier_R was used to predict sample subtype according to the Parker et al.^84^ subtype predictor. Second, a more recent classification model was applied using a robust classifier that integrates the PAM50 gene signature with Topological Data Analysis, resulting in seven subgroups with well-defined gene expression patterns^49^. The TDA classifier is based on the observed expression of five gene groups, basal (a), myo1 (b), myo2 (c), luminal (d), and Her2 (e) (Fig. 1a). The nomenclature of the identified TDA classes directly reflects the observed gene groups, for example, BasalHer2 samples are characterized by increased expression of the basal (a) and the Her2 (e) gene groups, and LumBasal samples by basal (a) and luminal (d) gene expression, and so on. An explanatory summary of the characteristics of the different TDA classes is included in Fig. 1a. Sample clustering according to both classification methods was visualized in a PAM50-based heatmap using the R package ComplexHeatmap (v1.20.0, ref. ^85^). Circos plots using the R package circlize (v0.4.6, ref. ^86^) depicted TDA reclassification of samples in comparison to PAM50 subtyping. The distribution of TDA subtypes within ancestries was assessed using stacked bar plots and *χ*^2^ tests.

### ICR consensus clustering

Consensus clustering of samples according to the expression values of 20 ICR genes was performed using the ConsensusClusterPlus (v1.42.0, ref. ^87^) R package with the following parameters: 5000 repeats, and agglomerative hierarchical clustering with ward criterion (Ward.D2) inner and complete outer linkage as previously described^51,88^. The optimal number of clusters for best segregation of samples was determined using the Calinski-Harabasz criterion with samples in intermediate clusters defined as “ICR Medium.” Samples of the TCGA dataset were clustered into three groups: ICR low (cluster 1), ICR medium (clusters 2 and 3), and ICR high (cluster 4). Due to the small number of samples, the RA-QA cohort was divided into 2 groups: ICR low (clusters 1, 2, and 3) and ICR high (cluster 4).

### Single-sample gene set enrichment analysis

Enrichment of specific gene sets, reflecting either abundance of immune cell populations or expression of tumor-related pathways, was defined by single-sample gene set enrichment analysis using R package GSVA (v.1.30.0, ref. ^89^)^90^. Gene set signatures of 24 distinct immune cell types or LES were used to deconvolute immune cell abundance^53^. Gene sets comprising numerous tumor-related pathways were obtained from multiple sources, including the Molecular Signatures Hallmark^91^ and Ingenuity Pathway Analysis (IPA) gene set collections and several signatures that have been associated with tumor immune escape^92–95^. Gene signature enrichment scores were compared based on ancestry using the two-tailed unpaired *t* test.

### XGBoost model

We utilized an optimized version of the white-box, nonlinear, ensemble gradient boosting machine called XGBoost to build our Cox regression model for survival analysis^96,97^. Gradient Boosting is a machine-learning technique based on a constructive strategy by which the learning procedure will additively fit new models, typically decision trees^98^ and repetitively leverage the patterns in residuals to provide a more accurate estimate of the response variable or time to event, that is, death in case of survival analysis. The patients who are alive are considered as right-censored, and since the XGBoost model takes only one label for the response variable as input, the censored survival information is converted to negative labels while performing the Cox proportional hazards modeling^99^. XGBoost is a scalable machine-learning technique for tree boosting, a learning technique to improve the regression performance of weak regressors by repeatedly adding new decision trees to the ensembles, which enhances performance in comparison to other boosting algorithms^96^. The main components of XGBoost algorithm are the objective function and its iterative solution. The objective function is initialized to describe the model’s performance. Given the training dataset, $D = \{ x^i,y^i\} _{i = 1}^N$, where *x*^*i*^ ∈ *R*^*d*^, *d* = 54, *y*^*i*^ ∈ *R*, *N* denotes the total number of training samples, *R* depicts the set of real numbers, and *D* represents the training set. The predicted output ${{\hat y}^l}$obtained from the ensemble model can be represented as: $\hat y^l = \mathop {\sum}\nolimits_{t = 1}^T {H_t} \left( {x^i} \right)$, where *H*_*t*_(*x*^*i*^) represents the prediction score of the *t*th decision tree for the *i*th patient in the training dataset. If the decision trees are allowed to grow unregulated, then the resulting model is bound to overfit^96^. Hence, the following objective has to be minimized:

1 $$J\left( H \right) = \mathop {\sum}\limits_{i = 1}^N {L\left( {y^i,\hat y^l} \right)} + \mathop {\sum}\limits_{t = 1}^T {\Omega \left( {H_t} \right)}$$

where *L* is the loss function and Ω() is the penalty that is used to prevent overfitting and is defined as $$\Omega \left( {H_t} \right) = \gamma A + \frac{1}{2}{\rlap{\kern.10em \hbox{$\bar{\,}$}}\raise-.32em\hbox{${{\lambda}}$}} \mathop {\sum}\nolimits_{j = 1}^A {w_j^2}$$, where *γ* and *ƛ* are the parameters that control the penalty for number of leaf nodes (*A*) and leaf weights (*w*), respectively, in the decision tree *H*_*t*_.

The objective function can be rewritten as $J\left( H \right) = {\mathop {\sum}\nolimits_{i = 1}^N} {L\left( {y^i, {{\hat y^l}}_{t - 1} + H_t\left( {x^i} \right)} \right)} + \mathop {\sum}\nolimits_{t = 1}^T {\Omega \left( {H_t} \right)}$. After applying a Taylor expansion^100^ and expanding Ω(*H*_*t*_), we obtain:

2 $${\it{J}}\left( {{\it{H}}_{\it{t}}} \right) = \mathop {\sum}\limits_{{\it{i}} = 1}^{\it{N}} {\left[ {{\it{g}}_{\it{i}}{\it{H}}_{\it{t}}\left( {{\it{x}}^{\it{i}}} \right) + \frac{1}{2}{\it{h}}_{\it{i}}^2{\it{H}}_{\it{t}}\left( {{\it{x}}^{\it{i}}} \right)} \right]} + \gamma {\it{A}} + \frac{1}{2}{\rlap{\kern.10em \hbox{$\bar{\,}$}}\raise-.32em\hbox{${{\lambda}}$}} \mathop {\sum}\limits_{j = 1}^{\it{A}} {{\it{w}}_j^2}$$

where $g_i = \partial _{y_{t - 1}}\left( {L\left( {y^i,\hat y_{t - 1}^l} \right)} \right)$ and $h_i = \partial _{y_{t - 1}}^2\left( {L\left( {y^i, {{\hat y^l}}_{t - 1} } \right)} \right)$ are the first- and second-order gradient statistics for the loss function *L*. For a fixed tree structure *H(x)*, where *I*_*j*_ = {*i*},∀*H*(*x*^*i*^) = *j* is an instance of leaf node *j*, the optimal weight $w_j^o$ for leaf node *j* is given by:

$${\it{w}}_{\it{j}}^{\it{o}} = \frac{{ - \mathop {\sum}\nolimits_{{\it{i}} \in {\it{I}}_{\it{j}}} {{\it{g}}_{\it{i}}} }}{{\mathop {\sum}\nolimits_{{\it{i}} \in {\it{I}}_{\it{j}}} {{\it{h}}_{\it{i}}} + {\rlap{\kern.10em \hbox{$\bar{\,}$}}\raise-.32em\hbox{${{\lambda}}$}} }}$$

The corresponding optimal objective function becomes:

3 $${\it{J}}\left( {{\it{H}}_{\it{t}}} \right) = \frac{{ - 1}}{2}\mathop {\sum}\limits_{{\it{j}} = 1}^{\it{A}} {\frac{{\left( {\mathop {\sum}\nolimits_{{\it{i}} \in {\it{I}}_{\it{j}}} {{\it{g}}_{\it{i}}} } \right)^2}}{{\left( {\mathop {\sum}\nolimits_{{\it{i}} \in {\it{I}}_{\it{j}}} {{\it{h}}_{\it{i}}} + {\rlap{\kern.10em \hbox{$\bar{\,}$}}\raise-.32em\hbox{${{\lambda}}$}} } \right)}}} + \gamma {\it{A}}$$

Equation 3 can be used as a scoring function to measure the quality of a tree structure *H*_*t*_ during iteration *t*. This score is equivalent to the impurity score used for evaluating decision trees in random forests^101^. We build our XGBoost model using the fast, greedy, and iterative algorithm by Chen et al.^96^ to identify the optimal tree structures.

### SHAP model

One of the disadvantages of the feature importance scores obtained from the XGBoost model is that the directionality is not apparent. For instance, when a particular pathway attains a high enrichment score, it is not clear whether this corresponds to a higher or lower risk of death. Moreover, at the test phase, it is a challenge for traditional white-box, tree-based, machine-learning techniques to provide information about the top five features driving the prediction to better or poorer survival prognosis. Recently, several techniques have been proposed to overcome aforementioned limitations, including LIME (Local Interpretable Model-agnostic Explanations)^102^ and SHAP^103^. These methods have the ability to interpret feature importance scores from complex training models and provide interpretable predictions for a test sample based on the top *k* features for that particular test instance. In our work, we used the SHAP method as it has been shown to outperform the LIME method and to be better aligned with human intuition^103^. The SHAP method is an additive feature attribution method where a test instance prediction is defined as a linear function of features that satisfies three critical properties: local accuracy, missingness, and consistency.

The explicit SHAP regression values are derived from a game-theory framework^104,105^ and can be computed as:

$$\Phi _i = \mathop {\sum}\limits_{S \subseteq Q - \{ i\} } {\frac{{\left| S \right|!\left( {\left| Q \right| - \left| S \right| - 1} \right)!}}{{\left| Q \right|!}}} \left[ {H_{S \cup \{ i\} }\left( {x_{S \cup \{ i\} }} \right) - H_S\left( {x_S} \right)} \right]$$

where *Q* represents the set of all *d* features, *S* represents the subsets obtained from *Q* after removing the *i*th feature, and *ɸ*_*i*_ is an estimate of the importance of feature *i* in the model. In order to refrain from undergoing 2^|*Q*|^ differences to estimate *ɸ*_*i*_, the SHAP method approximates the Shapley value by either performing Shapley sampling^106^ or Quantitative Input Influence^107^. A detailed description of model interpretation using the SHAP method has been outlined by Samek et al.^103^. In our work, SHAP values associated with a particular pathway in the XGBoost model provide information on the change in log (risk of death) for each feature of the Cox proportional hazards model.

### Survival analysis

Kaplan–Meier curves were generated using the ggsurvplot function from R package “survminer” (v0.4.8) to compare OS and DSS between ancestries, ICR clusters, and AMPK subgroups. Univariate Cox proportional hazards regression analysis was performed with the R package “survival.” AJCC pathologic tumor stage as described in the TCGA-CDR was used for stratified analysis within the BasalMyo class. Forest plots were generated using the R package forestplot (v1.7.2).

### Reporting summary

Further information on research design is available in the Nature Research Reporting Summary linked to this article.

## Supplementary information

Supplementary Figures

Reporting Summary Checklist

## Data Availability

The data generated and analyzed during this study are described in the following data record: 10.6084/m9.figshare.13379765^108^. The TCGA-BRCA cohort data are available through the GDC data portal (https://gdac.broadinstitute.org/runs/stddata__2016_01_28/data/BRCA/20160128/) or by using TCGA Assembler as detailed in the “Methods” section. TCGA Assembler is open source and freely available at http://www.compgenome.org/TCGA-Assembler/. The downloaded data product name is “illuminahiseq_rnaseqv2-RSEM_genes_normalized.” The RA-QA dataset RNA-sequencing data are openly available in fastq file format in the European Nucleotide Archive via the following accession: https://identifiers.org/ena.embl:PRJEB41828^109^. The RNA-seq Expression matrix, clinical data for the RA-QA cohort, and the enrichment scores data are openly available in figshare at 10.6084/m9.figshare.12901928^110^. Scripts used in the study can be found on Zenodo/github: 10.5281/zenodo.3707660^111^.

## Code availability

Information related to the data, models, and scripts of the XGBoost and SHAP models used in this manuscript can be found on zenodoo/github: 10.5281/zenodo.3707660.

## References

[1] Spratt DE (2016). Racial/ethnic disparities in genomic sequencing. JAMA Oncol..

[2] Newman LA (2017). Breast cancer disparities: socioeconomic factors versus biology. Ann. Surg. Oncol..

[3] Huo D (2009). Population differences in breast cancer: survey in indigenous African women reveals over-representation of triple-negative breast cancer. J. Clin. Oncol..

[4] Perez CA (2013). Black race as a prognostic factor in triple-negative breast cancer patients treated with breast-conserving therapy: a large, single-institution retrospective analysis. Breast Cancer Res. Treat..

[5] Komenaka IK (2010). Race and ethnicity and breast cancer outcomes in an underinsured population. J. Natl. Cancer Inst..

[6] Iqbal J, Ginsburg O, Rochon PA, Sun P, Narod SA (2015). Differences in breast cancer stage at diagnosis and cancer-specific survival by race and ethnicity in the United States. JAMA.

[7] Kroenke CH (2014). Race and breast cancer survival by intrinsic subtype based on PAM50 gene expression. Breast Cancer Res. Treat..

[8] Copson E (2014). Ethnicity and outcome of young breast cancer patients in the United Kingdom: the POSH study. Br. J. Cancer.

[9] Bowen RL, Duffy SW, Ryan DA, Hart IR, Jones JL (2008). Early onset of breast cancer in a group of British black women. Br. J. Cancer.

[10] Siddharth, S. & Sharma, D. Racial disparity and triple-negative breast cancer in african-american women: a multifaceted affair between obesity, biology, and socioeconomic determinants. *Cancers* **10**, 514 (2018). 10.3390/cancers10120514 PMC6316530 30558195

[11] Sweeney C (2014). Intrinsic subtypes from PAM50 gene expression assay in a population-based breast cancer cohort: differences by age, race, and tumor characteristics. Cancer Epidemiol. Biomark. Prev..

[12] Carey LA (2006). Race, breast cancer subtypes, and survival in the Carolina Breast Cancer Study. JAMA.

[13] Newman LA, Kaljee LM (2017). Health disparities and triple-negative breast cancer in African American women: a review. JAMA Surg..

[14] Newman LA, Reis-Filho JS, Morrow M, Carey LA, King TA (2015). The 2014 Society of Surgical Oncology Susan G. Komen for the Cure Symposium: triple-negative breast cancer. Ann. Surg. Oncol..

[15] Rapiti E (2017). Opportunities for improving triple-negative breast cancer outcomes: results of a population-based study. Cancer Med..

[16] Troester, M. A. et al. Racial differences in PAM50 subtypes in the Carolina Breast Cancer Study. *J. Natl. Cancer Inst*. **110**, 176–182 (2018). 10.1093/jnci/djx135 PMC6059138 28859290

[17] Killelea BK (2015). Racial differences in the use and outcome of neoadjuvant chemotherapy for breast cancer: results from the National Cancer Data Base. J. Clin. Oncol.

[18] Albain KS, Unger JM, Crowley JJ, Coltman CA, Hershman DL (2009). Racial disparities in cancer survival among randomized clinical trials patients of the Southwest Oncology Group. J. Natl. Cancer Inst..

[19] Newman LA (2006). Meta-analysis of survival in African American and white American patients with breast cancer: ethnicity compared with socioeconomic status. J. Clin. Oncol..

[20] Palmer JR (2013). Genetic susceptibility loci for subtypes of breast cancer in an African American population. Cancer Epidemiol. Biomark. Prev..

[21] Haddad SA (2016). An exome-wide analysis of low frequency and rare variants in relation to risk of breast cancer in African American Women: the AMBER Consortium. Carcinogenesis.

[22] Pitt JJ (2018). Characterization of Nigerian breast cancer reveals prevalent homologous recombination deficiency and aggressive molecular features. Nat. Commun..

[23] Ademuyiwa FO, Tao Y, Luo J, Weilbaecher K, Ma CX (2017). Differences in the mutational landscape of triple-negative breast cancer in African Americans and Caucasians. Breast Cancer Res. Treat..

[24] Grunda JM (2012). Differential expression of breast cancer-associated genes between stage- and age-matched tumor specimens from African- and Caucasian-American Women diagnosed with breast cancer. BMC Res. Notes.

[25] Martin DN (2009). Differences in the tumor microenvironment between African-American and European-American breast cancer patients. PLoS ONE.

[26] Field LA (2012). Identification of differentially expressed genes in breast tumors from African American compared with Caucasian women. Cancer.

[27] Stewart PA, Luks J, Roycik MD, Sang Q-XA, Zhang J (2013). Differentially expressed transcripts and dysregulated signaling pathways and networks in African American breast cancer. PLoS ONE.

[28] Lindner R (2013). Molecular phenotypes in triple negative breast cancer from African American patients suggest targets for therapy. PLoS ONE.

[29] Keenan T (2015). Comparison of the genomic landscape between primary breast cancer in African American versus white women and the association of racial differences with tumor recurrence. J. Clin. Oncol..

[30] Lehmann BD (2011). Identification of human triple-negative breast cancer subtypes and preclinical models for selection of targeted therapies. J. Clin. Invest..

[31] Gibbs LD, Vishwanatha JK (2017). Prognostic impact of AnxA1 and AnxA2 gene expression in triple-negative breast cancer. Oncotarget.

[32] Sugita B (2016). Differentially expressed miRNAs in triple negative breast cancer between African-American and non-Hispanic white women. Oncotarget.

[33] Lara OD (2020). Pan-cancer clinical and molecular analysis of racial disparities. Cancer.

[34] Nakshatri H, Anjanappa M, Bhat-Nakshatri P (2015). Ethnicity-dependent and -independent heterogeneity in healthy normal breast hierarchy impacts tumor characterization. Sci. Rep..

[35] Nalwoga H, Arnes JB, Wabinga H, Akslen LA (2010). Expression of aldehyde dehydrogenase 1 (ALDH1) is associated with basal-like markers and features of aggressive tumours in African breast cancer. Br. J. Cancer.

[36] Ginestier C (2007). ALDH1 is a marker of normal and malignant human mammary stem cells and a predictor of poor clinical outcome. Cell Stem Cell.

[37] Wend P (2013). WNT10B/β-catenin signalling induces HMGA2 and proliferation in metastatic triple-negative breast cancer. EMBO Mol. Med..

[38] Telonis AG, Rigoutsos I (2018). Race disparities in the contribution of miRNA isoforms and tRNA-derived fragments to triple-negative breast cancer. Cancer Res..

[39] Adams S (2014). Prognostic value of tumor-infiltrating lymphocytes in triple-negative breast cancers from two phase III randomized adjuvant breast cancer trials: ECOG 2197 and ECOG 1199. J. Clin. Oncol..

[40] Denkert C (2018). Tumour-infiltrating lymphocytes and prognosis in different subtypes of breast cancer: a pooled analysis of 3771 patients treated with neoadjuvant therapy. Lancet Oncol..

[41] Loi S (2013). Prognostic and predictive value of tumor-infiltrating lymphocytes in a Phase III Randomized Adjuvant Breast Cancer Trial in node-positive breast cancer comparing the addition of docetaxel to doxorubicin with doxorubicin-based chemotherapy: BIG 02–98. J. Clin. Oncol..

[42] Loi, S. et al. Tumor-infiltrating lymphocytes and prognosis: a pooled individual patient analysis of early-stage triple-negative breast cancers. *J. Clin. Oncol.*10.1200/JCO.18.01010 (2019). 10.1200/JCO.18.01010 PMC7010425 30650045

[43] Deshmukh SK (2015). Resistin and interleukin-6 exhibit racially-disparate expression in breast cancer patients, display molecular association and promote growth and aggressiveness of tumor cells through STAT3 activation. Oncotarget.

[44] Park N-J, Kang D-H (2013). Inflammatory cytokine levels and breast cancer risk factors: racial differences of healthy Caucasian and African American women. Oncol. Nurs. Forum.

[45] O’Meara T (2019). Immune microenvironment of triple-negative breast cancer in African-American and Caucasian women. Breast Cancer Res. Treat..

[46] Liu J (2018). An integrated TCGA Pan-Cancer Clinical Data resource to drive high-quality survival outcome analytics. Cell.

[47] Sayaman, R. W. et al. Germline genetic contribution to the immune landscape of cancer. *Immunity*. 10.1016/j.immuni.2021.01.011 (2021). 10.1016/j.immuni.2021.01.011 PMC8414660 33567262

[48] Carrot-Zhang J (2020). Comprehensive analysis of genetic ancestry and its molecular correlates in cancer. Cancer Cell.

[49] Mathews JC (2019). Robust and interpretable PAM50 reclassification exhibits survival advantage for myoepithelial and immune phenotypes. NPJ Breast Cancer.

[50] Huo D (2017). Comparison of breast cancer molecular features and survival by African and European ancestry in The Cancer Genome Atlas. JAMA Oncol..

[51] Hendrickx, W.et al. Identification of genetic determinants of breast cancer immune phenotypes by integrative genome-scale analysis. *Oncoimmunology* **6**, e1253654 (2017). 10.1080/2162402X.2016.1253654 PMC5353940 28344865

[52] Bedognetti D, Hendrickx W, Marincola FM, Miller LD (2015). Prognostic and predictive immune gene signatures in breast cancer. Curr. Opin. Oncol..

[53] Bindea G (2013). Spatiotemporal dynamics of intratumoral immune cells reveal the immune landscape in human cancer. Immunity.

[54] Bertucci F (2018). The immunologic constant of rejection classification refines the prognostic value of conventional prognostic signatures in breast cancer. Br. J. Cancer.

[55] Yoshihara, K. et al. Inferring tumour purity and stromal and immune cell admixture from expression data. *Nat. Commun*. **4**, 2612 (2013). 10.1038/ncomms3612 PMC3826632 24113773

[56] Chen, Y., Jia, Z., Mercola, D. & Xie, X. A Gradient Boosting Algorithm for survival analysis via direct optimization of concordance index. *Comput. Math. Methods Med.* **2013**, https://www.hindawi.com/journals/cmmm/2013/873595/ (2013). 10.1155/2013/873595 PMC3853154 24348746

[57] Nguyen, N. P. *Gradient Boosting for Survival Analysis with Applications in Oncology* (University of South Florida, 2020).

[58] Floares A (2017). The smallest sample size for the desired diagnosis accuracy. Int. J. Oncol. Cancer Ther..

[59] Rooney MS, Shukla SA, Wu CJ, Getz G, Hacohen N (2015). Molecular and genetic properties of tumors associated with local immune cytolytic activity. Cell.

[60] Gleason MX, Mdzinarishvili T, Sherman S (2012). Breast cancer incidence in black and white women stratified by estrogen and progesterone receptor statuses. PLoS ONE.

[61] Liu Z, Li M, Jiang Z, Wang X (2018). A comprehensive immunologic portrait of triple-negative breast cancer. Transl. Oncol..

[62] Denkert C (2010). Tumor-associated lymphocytes as an independent predictor of response to neoadjuvant chemotherapy in breast cancer. J. Clin. Oncol..

[63] Bracci L, Schiavoni G, Sistigu A, Belardelli F (2014). Immune-based mechanisms of cytotoxic chemotherapy: implications for the design of novel and rationale-based combined treatments against cancer. Cell Death Differ..

[64] de Kruijf EM (2010). The predictive value of HLA class I tumor cell expression and presence of intratumoral Tregs for chemotherapy in patients with early breast cancer. Clin. Cancer Res. Off..

[65] Bhattacharya, A. et al. A framework for transcriptome-wide association studies in breast cancer in diverse study populations. *Genome Biol*. **21**, 42, 10.1186/s13059-020-1942-6 (2020). 10.1186/s13059-020-1942-6 PMC7033948 32079541

[66] He Y, Jiang Z, Chen C, Wang X (2018). Classification of triple-negative breast cancers based on Immunogenomic profiling. J. Exp. Clin. Cancer Res..

[67] van der Weyden L (2017). Genome-wide in vivo screen identifies novel host regulators of metastatic colonization. Nature.

[68] Hadad SM (2009). Histological evaluation of AMPK signalling in primary breast cancer. BMC Cancer.

[69] Huang X (2016). High expressions of LDHA and AMPK as prognostic biomarkers for breast cancer. Breast.

[70] Cao W, Li J, Hao Q, Vadgama JV, Wu Y (2019). AMP-activated protein kinase: a potential therapeutic target for triple-negative breast cancer. Breast Cancer Res..

[71] Montero JC (2014). Active kinase profiling, genetic and pharmacological data define mTOR as an important common target in triple-negative breast cancer. Oncogene.

[72] Deng X-S (2012). Metformin targets Stat3 to inhibit cell growth and induce apoptosis in triple-negative breast cancers. Cell Cycle.

[73] Goodwin PJ (2011). Evaluation of metformin in early breast cancer: a modification of the traditional paradigm for clinical testing of anti-cancer agents. Breast Cancer Res. Treat..

[74] Attri KS, Murthy D, Singh PK (2017). Racial disparity in metabolic regulation of cancer. Front. Biosci. Landmark Ed..

[75] Tayyari F (2018). Metabolic profiles of triple-negative and luminal A breast cancer subtypes in African-American identify key metabolic differences. Oncotarget.

[76] Shen J, Yan L, Liu S, Ambrosone CB, Zhao H (2013). Plasma metabolomic profiles in breast cancer patients and healthy controls: by race and tumor receptor subtypes. Transl. Oncol..

[77] Zhu Y, Qiu P, Ji Y (2014). TCGA-Assembler: an open-source pipeline for TCGA data downloading, assembling, and processing. Nat. Methods.

[78] Thorsson V (2018). The immune landscape of cancer. Immunity.

[79] Dodt M, Roehr JT, Ahmed R, Dieterich C (2012). FLEXBAR—Flexible Barcode and Adapter Processing for Next-Generation Sequencing Platforms. Biology.

[80] Kim D, Langmead B, Salzberg SL (2015). HISAT: a fast spliced aligner with low memory requirements. Nat. Methods.

[81] Liao, Y., Smyth, G. K. & Shi, W. The R package Rsubread is easier, faster, cheaper and better for alignment and quantification of RNA sequencing reads. *Nucleic Acids Res*. **47**, e47, http://subread.sourceforge.net/ (2019). 10.1093/nar/gkz114 PMC6486549 30783653

[82] Risso D, Schwartz K, Sherlock G, Dudoit S (2011). GC-content normalization for RNA-seq data. BMC Bioinform..

[83] Ben Bolstad. <bmb at bmbolstad.com>. preprocessCore: a collection of pre-processing functions. Bioconductor version: Release (3.9). 10.18129/B9.bioc.preprocessCore (2019).

[84] Parker JS (2009). Supervised risk predictor of breast cancer based on intrinsic subtypes. J. Clin. Oncol..

[85] Gu Z, Eils R, Schlesner M (2016). Complex heatmaps reveal patterns and correlations in multidimensional genomic data. Bioinformatics.

[86] Gu Z, Gu L, Eils R, Schlesner M, Brors B (2014). circlize implements and enhances circular visualization in R. Bioinformatics.

[87] Wilkerson MD, Hayes DN (2010). ConsensusClusterPlus: a class discovery tool with confidence assessments and item tracking. Bioinformatics.

[88] Roelands, J. et al. Oncogenic state sdictate the prognostic and predictive connotations of intratumoral immune response. *J Immunother Cancer*. e000617, 10.1136/jitc-2020-000617 (2020). 10.1136/jitc-2020-000617 PMC7223637 32376723

[89] Hänzelmann S, Castelo R, Guinney J (2013). GSVA: gene set variation analysis for microarray and RNA-seq data. BMC Bioinform..

[90] Barbie DA (2009). Systematic RNA interference reveals that oncogenic KRAS-driven cancers require TBK1. Nature.

[91] Liberzon A (2015). The Molecular Signatures Database Hallmark gene set collection. Cell Syst..

[92] Bedognetti D, Roelands J, Decock J, Wang E, Hendrickx W (2017). The MAPK hypothesis: immune-regulatory effects of MAPK-pathway genetic dysregulations and implications for breast cancer immunotherapy. Emerg. Top. Life Sci..

[93] Miller, L. D. et al. Immunogenic subtypes of breast cancer delineated by gene classifiers of immune responsiveness. *Cancer Immunol. Res.*10.1158/2326-6066.CIR-15-0149 (2016). 10.1158/2326-6066.CIR-15-0149 PMC4930674 27197066

[94] Salerno EP (2016). Human melanomas and ovarian cancers overexpressing mechanical barrier molecule genes lack immune signatures and have increased patient mortality risk. Oncoimmunology.

[95] Lu R, Turan T, Samayoa J, Marincola FM (2017). Cancer immune resistance: can theories converge?. Emerg. Top. Life Sci..

[96] Chen, T. & Guestrin, C. XGBoost: a scalable tree boosting system. In *Proceedings of the 22nd ACM SIGKDD International Conference on Knowledge Discovery and Data Mining (KDD’16*). Association for Computing Machinery, New York, NY, USA, 785–794, 10.1145/2939672.2939785 (2016).

[97] Friedman JH (2001). Greedy Function Approximation: a gradient boosting machine. Ann. Stat..

[98] Quinlan JR (1999). Simplifying decision trees. Int. J. Hum. Comput. Stud..

[99] Crichton N (2002). Cox proportional hazards model. J. Clin. Nurs..

[100] Roy, R. in *Pi: A Source Book* (eds Berggren, L., Borwein, J. & Borwein, P.) 92–107 (Springer, 1997).

[101] Breiman L (2001). Random forests. Mach. Learn..

[102] Ribeiro, M., Singh, S. & Guestrin, C. Why should i trust you?: explaining the predictions of any classifier. In *Proceedings of the 22nd ACM SIGKDD International Conference on Knowledge Discovery and Data Mining*, 1135–1144 (ACM, 2016).

[103] Samek, W., Montavon, G., Vedaldi, A., Hansen, L. & Muller, K. *Explainable AI: Interpreting, Explaining and Visualizing Deep Learning|Wojciech Samek* (Springer, 2019).

[104] Lipovetsky S, Conklin M (2001). Analysis of regression in game theory approach. Appl. Stoch. Models Bus. Ind..

[105] Kuhn, H. & Tucker, A. *Contributions to the Theory of Games (AM-28)*, Vol. II (Princeton University Pres, 1953).

[106] Strumbelj E, Kononenko I (2014). Explaining prediction models and individual predictions with feature contributions. Knowl. Inf. Syst..

[107] Datta, A., Sen, S. & Zick, Y. Algorithmic transparency via quantitative input influence: theory and experiments with learning systems. In *2016 IEEE Symposium on Security and Privacy (SP)*, 598–617 (IEEE, 2016).

[108] Roelands, J. et al. Metadata record for the manuscript: ancestry-associated transcriptomic profiles of breast cancer in patients of African, Arab and European ancestry. figshare 10.6084/m9.figshare.13379765 (2020). 10.1038/s41523-021-00215-x PMC7870839 33558495

[109] European Nucleotide Archive. https://identifiers.org/ena.embl:PRJEB41828 (2020).

[110] Roelands, J. et al. Data supporting the manuscript: ancestry-associated transcriptomic profiles of breast cancer in patients of African, Arab and European ancestry. figshare 10.6084/m9.figshare.12901928 (2020). 10.1038/s41523-021-00215-x PMC7870839 33558495

[111] Roelands, J. & Hendrickx, W. Zenodo. 10.5281/zenodo.3707660 (2020).

