# Supplementary material for: Ancestry-associated transcriptomic profiles of breast cancer in patients of African, Arab, and European ancestry
Source: NPJ Breast Cancer. 2021 Feb 8;7:10. doi: 10.1038/s41523-021-00215-x (PMC7870839; doi:10.1038/s41523-021-00215-x)
Supplement: Supplementary file 1 — Supplementary Figures [file 41523_2021_215_MOESM1_ESM.pdf]

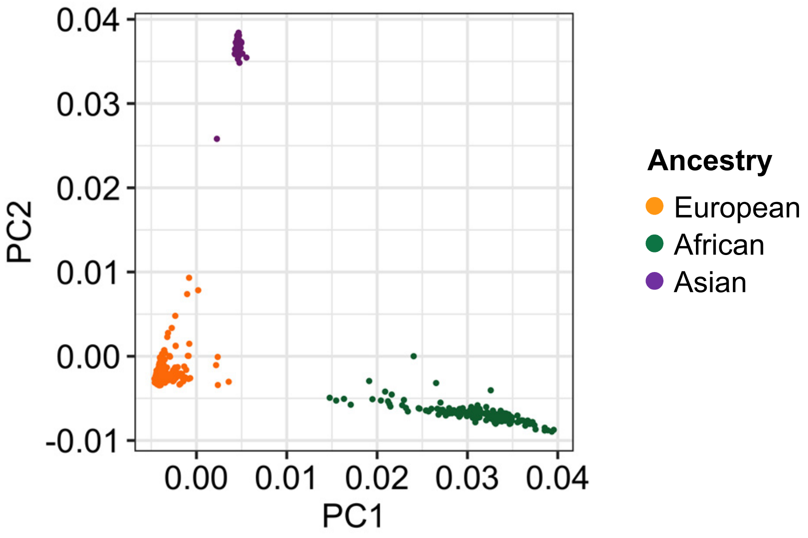

Supplementary Figure 1. Principal Component Analysis (PCA) of genotyping array identifies major clusters of continental ancestry. PC1 versus PC2 of the TCGA BRCA cohort annotated by SNP-based inference of ancestry from Jian Carrot-Zhang *et al* [48].

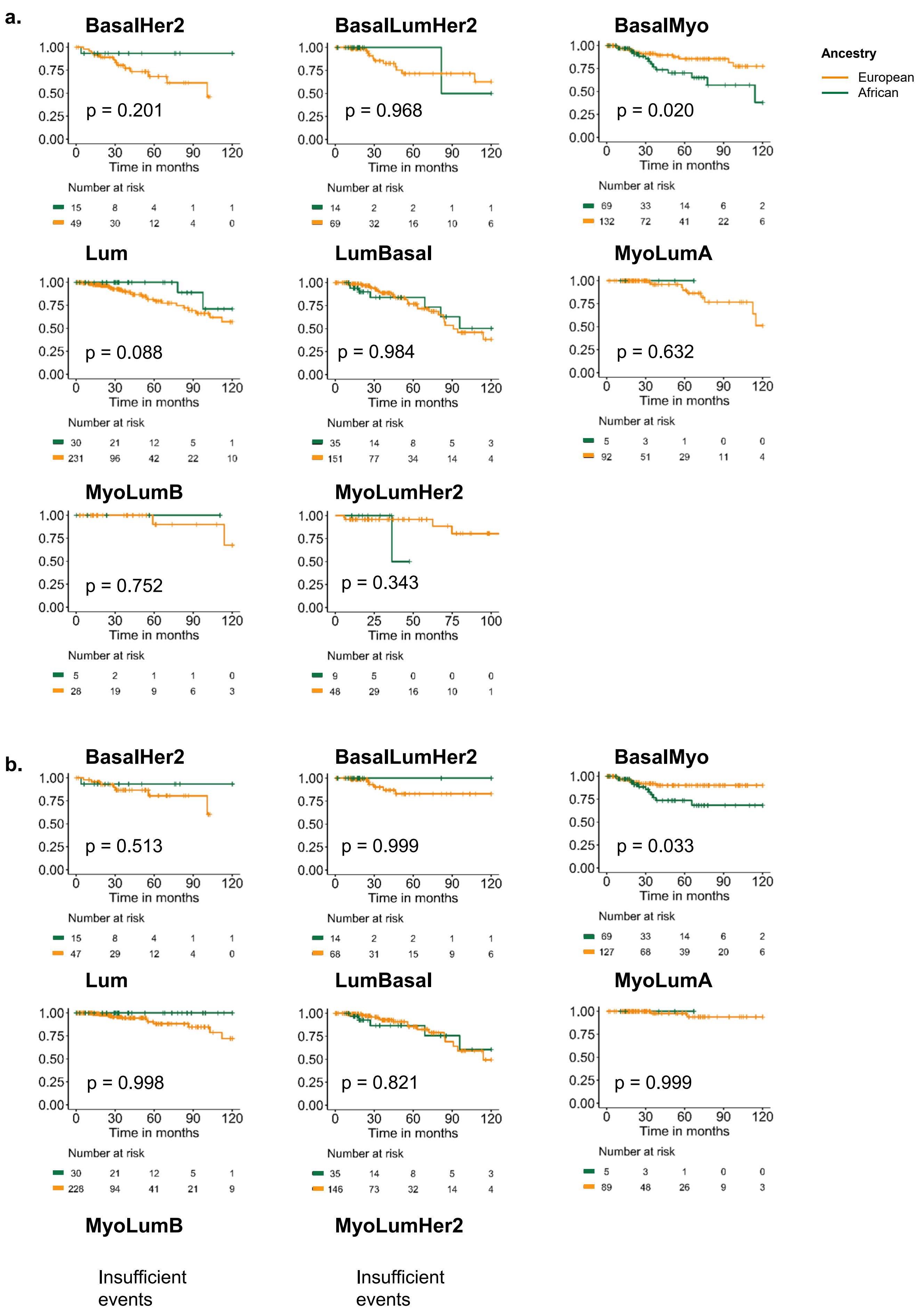

Supplementary Figure 2. Survival analysis of TDA subtypes by ancestry. Kaplan Meier plots showing **a.** overall survival and **b.** disease specific survival by ancestry within each TDA breast cancer subtype.

a.

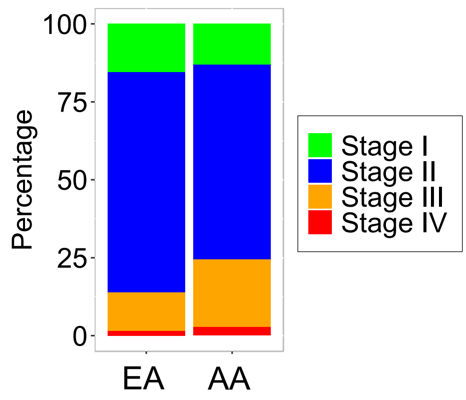

	Stage I and II	Stage III and IV	
EA	111 (86.0%)	18 (14.0%)	129
AA	52 (75.4%)	17 (24.6%)	69

X-squared = 2.8304, df = 1, p-value = 0.09249

b.

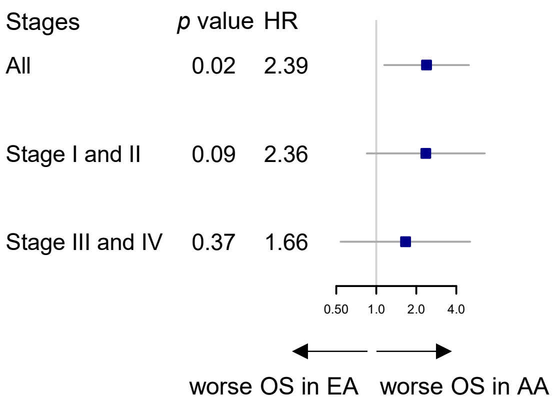

c. **All patients**

Variables	Univariate HR (95% CI)	*p*	Multivariate HR (95% CI)	*p*
Ancestry (AA vs EA)	2.394 (1.15- 4.984)	0.0196 *	2.089 (0.9763- 4.47)	0.0577
Stage (III&IV vs I&II)	5.296 (2.516- 11.15)	1.14e-05 ***	4.720 (2.2197- 10.04)	5.54e-05 ***
Ancestry (AA vs EA)	2.394 (1.15- 4.984)	0.0196 *	1.966 (0.9121- 4.239)	0.0845
Stage (III&IV vs I&II)	5.296 (2.516- 11.15)	1.14e-05 ***	4.897 (2.2913- 10.466)	4.14e-05 ***
Age (continuous)	1.021 (0.9913- 1.052)	0.167	1.021 (0.9931- 1.050)	0.1417

BasalMyo Medium + Low

Variables	Univariate HR (95% CI)	*p*	Multivariate HR (95% CI)	*p*
Ancestry (AA vs EA)	2.502 (1.092-5.731)	0.0302 *	2.433 (1.018-5.813)	0.045369 *
Stage (III&IV vs I&II)	4.943 (2.138-11.43)	1.87e-04 ***	4.514 (1.941- 10.496)	4.65e-04 ***
Ancestry (AA vs EA)	2.502 (1.092-5.731)	0.0302 *	2.361 (0.9755-5.713)	0.056782
Stage (III&IV vs I&II)	4.943 (2.138-11.43)	1.87e-04 ***	4.408 (1.8765-10.356)	6.63e-04 ***
Age (continuous)	1.022 (0.9826- 1.063)	0.278	1.007 (0.9732-1.043)	0.673247

Supplementary Figure 3. Distribution of pathological stage by ancestry. a. Stacked barchart of AJCC pathological stages in BasalMyo tumors of EA and AA patients (upper panel). Contingency table of stage by ancestry and corresponding Chi-squared statistics (lower panel). **b.** Cox proportional hazard survival analysis of ancestry in all stages, and stratified by early stage (Stage I and II) and advanced stage (Stage III and IV). HR and corresponding 95%-confidence intervals are indicated. **c.** Univariate and multivariate analysis. * p<0.05, *** p<0.001

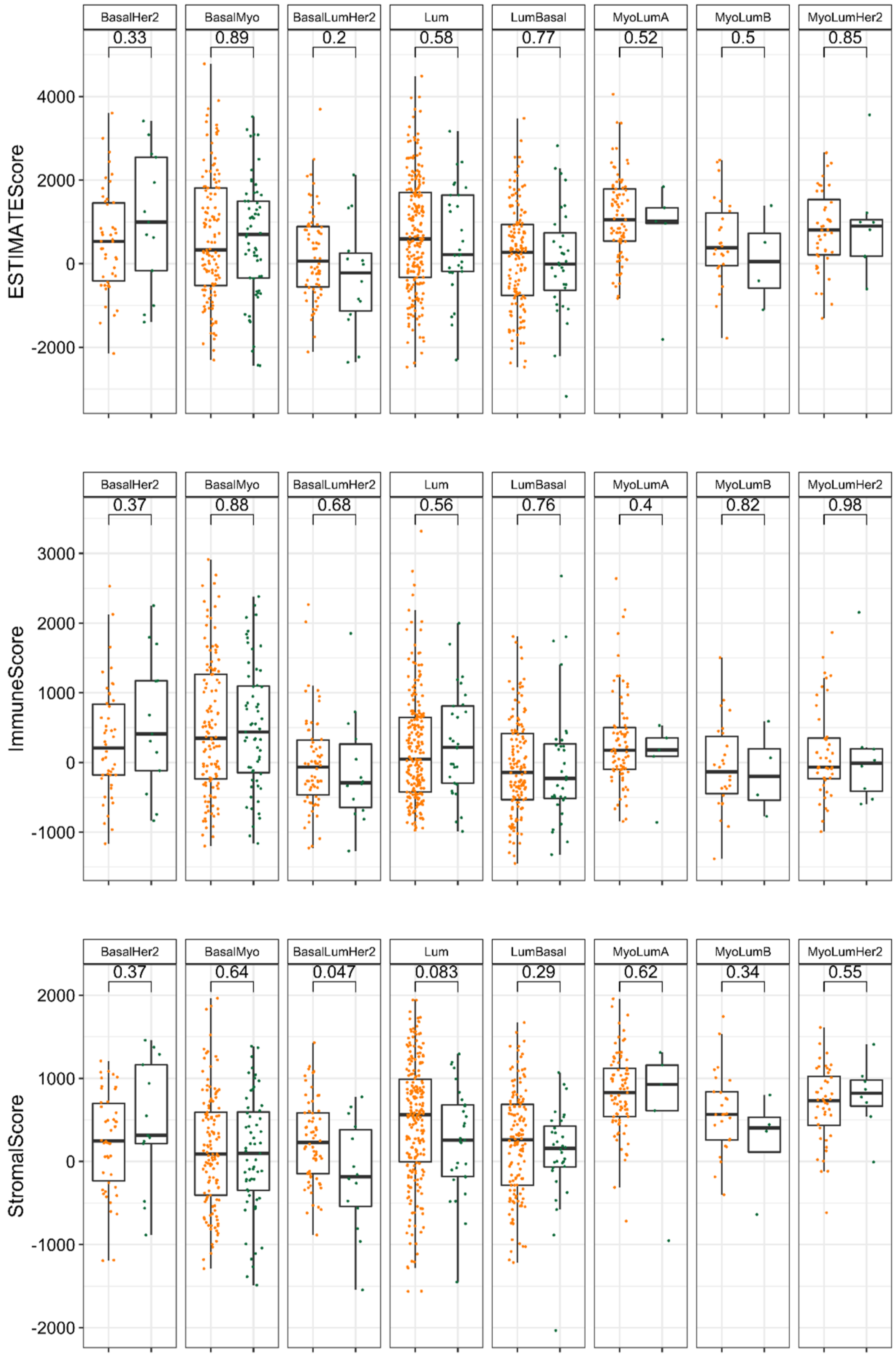

Supplementary Figure 4. Tumor cellularity and cellular composition by ancestry. ESTIMATE score (*upper panels*), ESTIMATE ImmuneScore (*middle panels*), and ESTIMATE StromalScore (*lower panels*) by ancestry within TDA breast cancer subtypes. p-values determined by two-tailed t-test. Box plots indicate medians and interquartile range, whiskers represent 10th and 90th percentile. All data points are plotted individually.

All patients (n= 1082)

Cell type	*p* value	FDR	HR
T helper cells	0.0011	0.025	0.0866
CD8 T cells	0.01	0.058	0.146
Cytotoxic cells	0.0025	0.029	0.172
B cells	0.032	0.11	0.184
T cells	0.0064	0.049	0.189
TFH	0.021	0.082	0.194
NK cells	0.055	0.14	0.3
ICR	0.014	0.064	0.306
NK CD56dim cells	0.067	0.15	0.381
Eosinophils	0.051	0.14	0.384
NK CD56bright cells	0.18	0.35	0.458
aDC	0.13	0.27	0.481
TReg	0.36	0.61	0.574
Neutrophils	0.37	0.61	0.575
pDC	0.54	0.68	0.585
Th1 cells	0.43	0.65	0.636
DC	0.53	0.68	0.664
iDC	0.58	0.7	0.726
Tcm	0.67	0.74	0.726
Tem	0.63	0.72	0.761
Th17 cells	0.93	0.98	0.96
Macrophages	0.98	0.98	0.982
Th2 cells	0.45	0.65	1.44

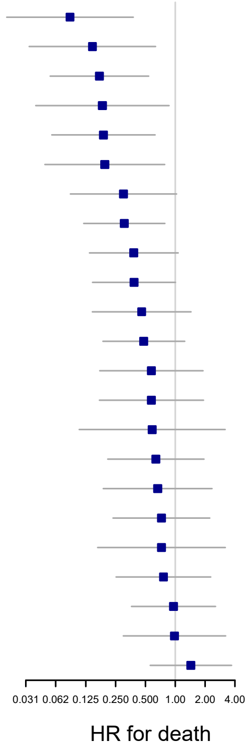

EA (n= 811)

Cell type	*p* value	FDR	HR
T helper cells	0.00046	0.011	0.0427
CD8 T cells	0.06	0.2	0.193
Cytotoxic cells	0.017	0.078	0.203
B cells	0.0059	0.045	0.057
T cells	0.0043	0.045	0.116
TFH	0.012	0.071	0.117
NK cells	0.37	0.78	0.516
ICR	0.048	0.19	0.334
NK CD56dim cells	0.086	0.25	0.346
Eosinophils	0.35	0.78	0.582
NK CD56bright cells	0.91	0.97	0.928
aDC	0.33	0.78	0.577
TReg	0.66	0.97	0.731
Neutrophils	0.92	0.97	1.07
pDC	0.97	0.97	0.966
Th1 cells	0.79	0.97	0.838
DC	0.96	0.97	0.967
iDC	0.65	0.97	1.35
Tcm	0.8	0.97	1.26
Tem	0.82	0.97	1.16
Th17 cells	0.64	0.97	1.31
Macrophages	0.81	0.97	1.17
Th2 cells	0.65	0.97	1.27

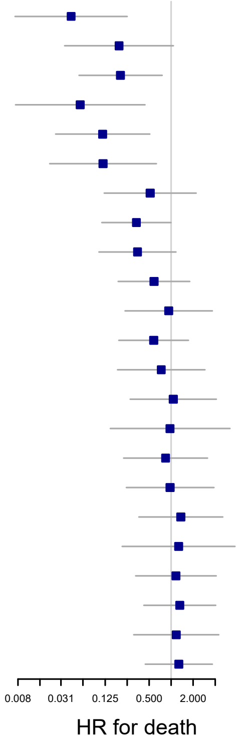

AA (n= 184)

Cell type	*p* value	FDR	HR
T helper cells	0.68	0.75	0.557
CD8 T cells	0.17	0.39	0.107
Cytotoxic cells	0.11	0.38	0.137
B cells	0.99	0.99	1.02
T cells	0.63	0.72	0.589
TFH	0.53	0.67	0.433
NK cells	0.17	0.39	0.122
ICR	0.21	0.45	0.279
NK CD56dim cells	0.73	0.77	0.684
Eosinophils	0.1	0.38	0.185
NK CD56bright cells	0.059	0.27	0.0849
aDC	0.26	0.47	0.314
TReg	0.44	0.6	0.378
Neutrophils	0.0079	0.18	0.0234
pDC	0.17	0.39	0.0677
Th1 cells	0.26	0.47	0.284
DC	0.051	0.27	0.0337
iDC	0.054	0.27	0.0915
Tcm	0.42	0.6	0.299
Tem	0.042	0.27	0.078
Th17 cells	0.3	0.5	0.317
Macrophages	0.34	0.53	0.268
Th2 cells	0.59	0.71	2.02

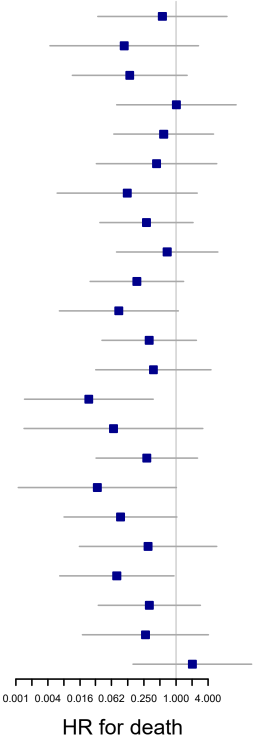

Supplementary Figure 5. Prognostic significance of leukocyte subgroup enrichment scores by ancestry. Forest plots showing HRs for death (overall survival) and corresponding 95%-confidence interval for single sample gene set enrichment scores of immune cell type-specific gene signatures, across all patients (left), and within EA (middle) and AA (right) subgroups. Signatures are ordered by ascending HR in all patients. False Discovery Rate (FDR) was calculated by Benjamini-Hochberg method.

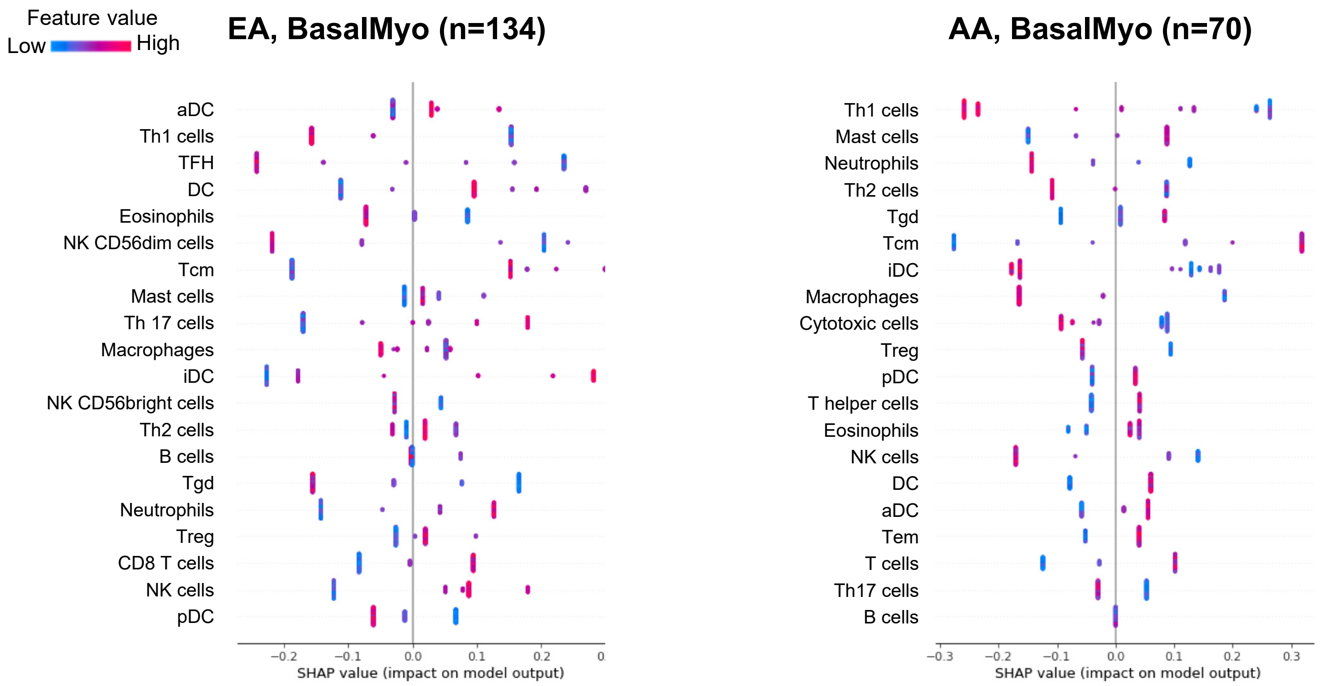

Supplementary Figure 6. SHAP plots of leukocyte subgroup enrichment scores in BasalMyo tumors by ancestry. Immune cell subsets are ranked by p-value to reflect the importance of each feature in the survival model. Each dot represents a single sample and is colored by relative enrichment score. Corresponding impact on model output (SHAP value) ranges from -1 (indicating absence of an event) to +1 (indicating occurrence of an event, in this case death).

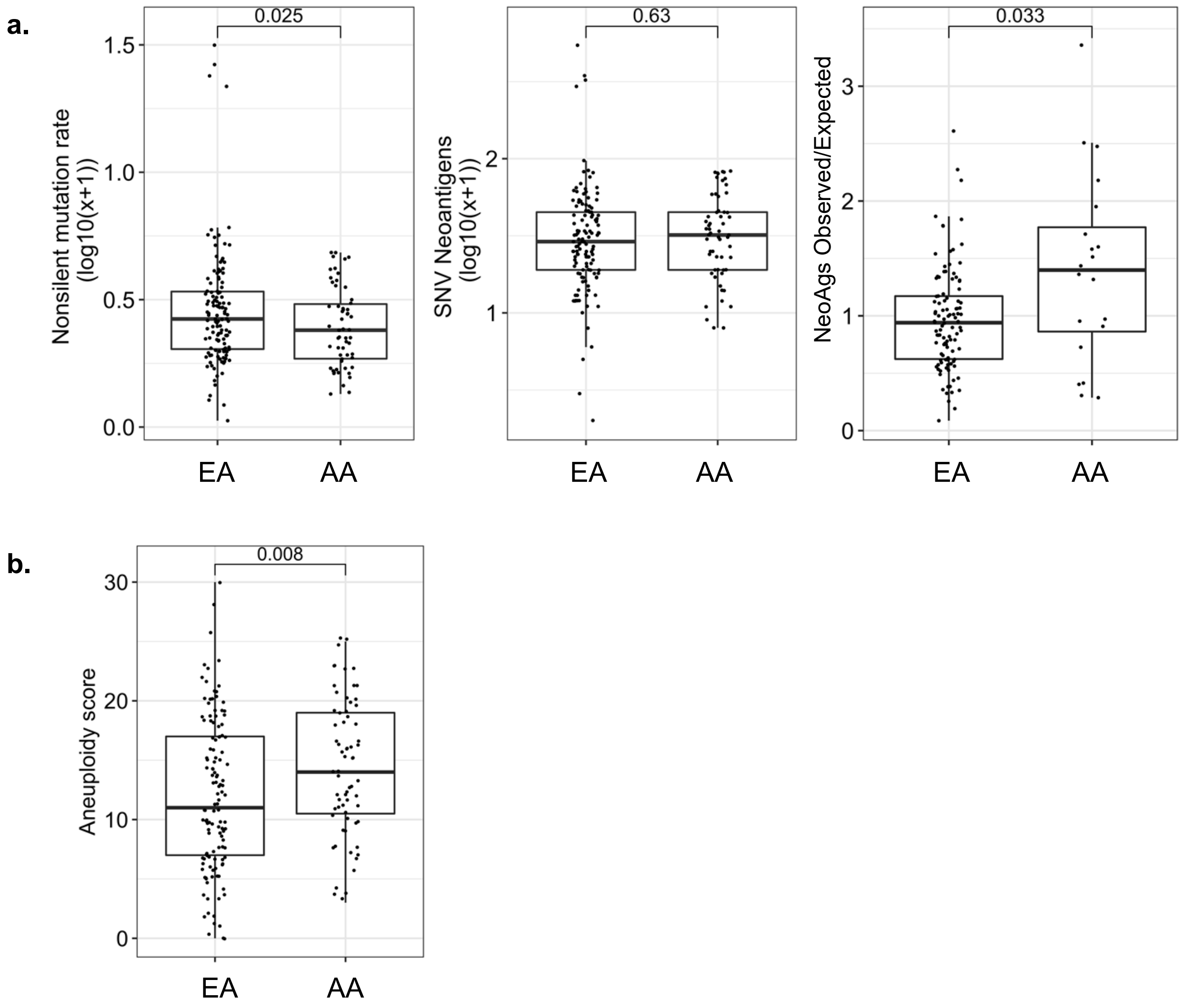

Supplementary Figure 7. Genomic aberrations in BasalMyo tumors by ancestry. a. Boxplot of non-silent mutation rate (log10 transformed with +1 offset) (*left*), SNV neoantigens (log10 transformed with +1 offset), and ratio between observed and expected neoantigens [58] by ancestry. **b.** Boxplot of aneuploidy score by ancestry. p-values determined by two-tailed t-test. Box plots indicate medians and interquartile range, whiskers represent 10th and 90th percentile. All data points are plotted individually. SNV, single nucleotide variant.
