# Supplementary material for: Ancestry-associated transcriptomic profiles of breast cancer in patients of African, Arab, and European ancestry
Source: NPJ Breast Cancer. 2021 Feb 8;7:10. doi: 10.1038/s41523-021-00215-x (PMC7870839; doi:10.1038/s41523-021-00215-x)
Supplement: Supplementary file 2 — Reporting Summary Checklist [file 41523_2021_215_MOESM2_ESM.pdf]

nature research

Corresponding author(s): Julie Decock, Wouter Hendrickx, Davide Bedognetti

Last updated by author(s): 2020/12/01

Reporting Summary

Nature Research wishes to improve the reproducibility of the work that we publish. This form provides structure for consistency and transparency in reporting. For further information on Nature Research policies, see our Editorial Policies and the Editorial Policy Checklist.

Statistics

For all statistical analyses, confirm that the following items are present in the figure legend, table legend, main text, or Methods section.

n/a	Confirmed	
☐	☒	The exact sample size (*n*) for each experimental group/condition, given as a discrete number and unit of measurement
☒	☐	A statement on whether measurements were taken from distinct samples or whether the same sample was measured repeatedly
☐	☒	The statistical test(s) used AND whether they are one- or two-sided<br>*Only common tests should be described solely by name; describe more complex techniques in the Methods section.*
☐	☒	A description of all covariates tested
☐	☒	A description of any assumptions or corrections, such as tests of normality and adjustment for multiple comparisons
☐	☒	A full description of the statistical parameters including central tendency (e.g. means) or other basic estimates (e.g. regression coefficient) AND variation (e.g. standard deviation) or associated estimates of uncertainty (e.g. confidence intervals)
☒	☐	For null hypothesis testing, the test statistic (e.g. *F*, *t*, *r*) with confidence intervals, effect sizes, degrees of freedom and *P* value noted<br>*Give P values as exact values whenever suitable.*
☒	☐	For Bayesian analysis, information on the choice of priors and Markov chain Monte Carlo settings
☒	☐	For hierarchical and complex designs, identification of the appropriate level for tests and full reporting of outcomes
☒	☐	Estimates of effect sizes (e.g. Cohen's *d*, Pearson's *r*), indicating how they were calculated

Our web collection on statistics for biologists contains articles on many of the points above.

Software and code

Policy information about availability of computer code

Data collection	For TCGA data collection, TCGA Assembler (v.2.0.3) was used to download the RNASeq data as described in detail in the Methods section. Corresponding R code for both TCGA and RAQA cohort analysis is available via GitHub as stated in the the data availability statement.
Data analysis	All R scripts for analysis of the BRCA-TCGA as well as the RA-QA are shared via GitHub. Information related to the data, models and scripts of the XGBoost and SHAP models is shared at the same location.

For manuscripts utilizing custom algorithms or software that are central to the research but not yet described in published literature, software must be made available to editors and reviewers. We strongly encourage code deposition in a community repository (e.g. GitHub). See the Nature Research guidelines for submitting code & software for further information.

Data

Policy information about availability of data

All manuscripts must include a data availability statement. This statement should provide the following information, where applicable:

- Accession codes, unique identifiers, or web links for publicly available datasets
- A list of figures that have associated raw data
- A description of any restrictions on data availability

TCGA-BRCA cohort is available through GDC data portal or by using the R script detailed in the method section. Data pertaining to the RA-QA cohort can be downloaded via figshare: 10.6084/m9.figshare.12901928. Scripts used in this manuscript can be found on zenodoo/github : 10.5281/zenodo.3707660. Information related to the data, models and scripts of the XGBoost and SHAP models used in this manuscript can be found on zenodoo/github : 10.5281/zenodo.3707660.

April 2020

Field-specific reporting

Please select the one below that is the best fit for your research. If you are not sure, read the appropriate sections before making your selection.

☒ Life sciences ☐ Behavioural & social sciences ☐ Ecological, evolutionary & environmental sciences

For a reference copy of the document with all sections, see nature.com/documents/nr-reporting-summary-flat.pdf

Life sciences study design

All studies must disclose on these points even when the disclosure is negative.

Sample size	No sample size calculation was performed. We included all breast cancer patients from the TCGA breast cancer dataset for which ancestry was determined. With regards to the RA-QA cohort, we enrolled all female breast cancer patients with available tumor tissues that were newly diagnosed between 2004-2010. The RA-QA study protocol was granted waiver of informed consent under the condition of anonymization and no additional intervention for the participants.
Data exclusions	We excluded 31 patients from our ancestry-based analyses. Firstly, 16 patients with American inferred ancestry as the number of samples in this cluster is limited as well as one patient who self-identified as not Hispanic or Latino. Secondly, six patients without self-reported or inferred ancestry, and thirdly exceptional cases of discordance between self-reported and SNP-based ancestry (n=8; 0.9%) were excluded.
Replication	At the moment, the TCGA dataset represents the only large-scale dataset that is available with ancestry information and extensive molecular profiling. Replication of these analyses in an independent cohort would be very valuable, although not possible at the moment.
Randomization	Randomization was not applicable for this study. Where appropriate, multivariate analyses were performed with adjustment for age and pathological tumor stage.
Blinding	Blinding was not applicable for this specific study. All data analyses were systematically performed to compare transcriptomic differences between ancestries.

Reporting for specific materials, systems and methods

We require information from authors about some types of materials, experimental systems and methods used in many studies. Here, indicate whether each material, system or method listed is relevant to your study. If you are not sure if a list item applies to your research, read the appropriate section before selecting a response.

Materials & experimental systems

n/a	Involved in the study	
☒	☐	Antibodies
☒	☐	Eukaryotic cell lines
☒	☐	Palaeontology and archaeology
☒	☐	Animals and other organisms
☒	☐	Human research participants
☒	☐	Clinical data
☒	☐	Dual use research of concern

Methods

n/a	Involved in the study	
☒	☐	ChIP-seq
☒	☐	Flow cytometry
☒	☐	MRI-based neuroimaging
